# Competitive Adsorption of Substrate and Solvent in Sn‐Beta Zeolite During Sugar Isomerization

**DOI:** 10.1002/cssc.201600800

**Published:** 2016-10-28

**Authors:** William N. P. van der Graaff, Christiaan H. L. Tempelman, Guanna Li, Brahim Mezari, Nikolay Kosinov, Evgeny A. Pidko, Emiel J. M. Hensen

**Affiliations:** ^1^ Inorganic Materials Chemistry GroupSchuit Institute of CatalysisEindhoven University of TechnologyPO Box 5135600 MBEindhovenThe Netherlands; ^2^ Institute for Complex Molecular SystemsEindhoven University of TechnologyEindhovenThe Netherlands; ^3^ Theoretical Chemistry groupLaboratory for Solution Chemistry of Advanced Materials and TechnologiesITMO UniversitySt. PetersburgRussia

**Keywords:** carbohydrates, competitive adsorption, Lewis acids, Sn-beta, solvent replacement

## Abstract

The isomerization of 1,3‐dihydroxyactone and d‐glucose over Sn‐Beta zeolite was investigated by in situ ^13^C NMR spectroscopy. The conversion rate at room temperature is higher when the zeolite is dehydrated before exposure to the aqueous sugar solution. Mass transfer limitations in the zeolite micropores were excluded by comparing Sn‐Beta samples with different crystal sizes. Periodic density functional theory (DFT) calculations show that sugar and water molecules compete for adsorption on the active framework Sn centers. Careful solvent selection may thus increase the rate of sugar isomerization. Consistent with this prediction, batch catalytic experiments show that the use of a co‐solvent, such as tetrahydrofuran, that strongly interacts with the Sn centers suppresses glucose isomerization. On the other hand, the use of ethanol as cosolvent results in significantly higher isomerization activity in comparison with pure water because of decreased competition with glucose adsorption on zeolitic Sn sites.

Glucose is the cheapest of all hexose sugars and can, in contrast to fructose and mannose, be obtained in relatively pure form from lignocellulosic biomass. Isomerization of glucose into fructose is important for two reasons: 1) catalyzed by glucose isomerase, this reversible reaction is used at the industrial scale to produce high‐fructose corn syrup; 2) in addition, the aldose‐ketose isomerization is at the center of the attention of the scientific community in the context of biomass valorization into fuels and chemicals, as fructose can be readily dehydrated in high yield to 5‐hydroxymethylfurfural, a prospective platform molecule.^1^, ^2^, ^3^, ^4^ For implementation of glucose isomerization in biorefineries, enzymatic isomerization needs to be replaced by more active and robust catalyst systems. Glucose isomerization by bases is known, but suffers from by‐product formation and waste issues.^5^ Recently, Lewis acids have been identified as the preferred chemocatalysts for glucose isomerization.^6^, ^7^, ^8^, ^9^

From these studies, Sn‐Beta zeolite has emerged as particularly effective for catalyzing carbohydrate transformations. Sn‐Beta can isomerize hexoses, pentoses, and trioses by intramolecular hydride and carbon shift reactions in various solvents.^8^, ^10^, ^11^, ^12^ Another platform molecule is lactic acid, which can be obtained from fructose via retro‐aldol condensation^13^, ^14^ and hydride shift reactions involving 1,3‐dihydroxyacetone and glyceraldehyde as intermediates (Scheme 1). Earlier, Sn‐Beta has shown its promise in catalyzing Meerwein–Ponndorf–Verley reactions^15^ between alcohols and ketones as well as Baeyer–Villiger oxidation of cyclic ketones.^16^

**Scheme 1:**
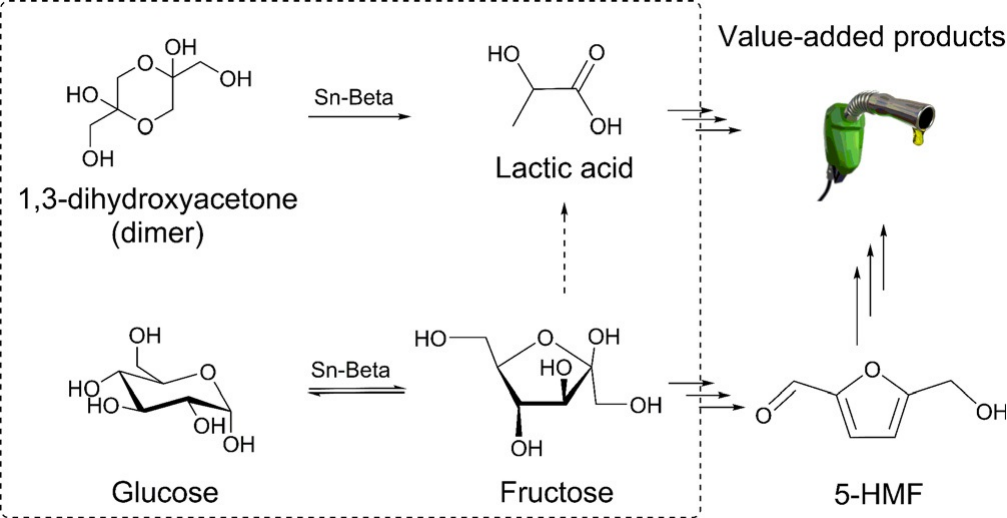
Sn‐Beta‐catalyzed isomerization of triose (1,3‐dihydroxyacetone) and hexose (glucose) in the framework of biomass valorization.

It is well known that the Lewis acidic Sn sites embedded in the microporous framework of Beta zeolite play a key role in these applications and their function resembles to some extent the action of enzymes. Still, the activity of Sn‐Beta in sugar isomerization is low compared with enzymes. Although it is well known that slow diffusion in zeolitic micropores can limit the reaction rate,^17^, ^18^, ^19^, ^20^, ^21^, ^22^, ^23^, ^24^ such effects have not been reported for Sn‐Beta‐catalyzed isomerization of sugars.^25^

Herein, we report that Sn‐Beta is already effective at room temperature as a catalyst for the isomerization of 1,3‐dihydroxyacetone (DHA) and d‐glucose. We followed the isomerization of C3 and C6 sugars in Sn‐Beta by in situ ^13^C NMR spectroscopy and noted pronounced activity differences between dehydrated and hydrated zeolites exposed to an aqueous sugar solution. We confirm that the catalytic conversion of sugars in water is slow owing to sluggish displacement of solvent molecules coordinating to active Sn centers by sugar molecules. This insight allowed optimizing the solvent system towards improved isomerization activity. The results attest to the importance of competitive adsorption of sugars and polar solvent molecules in sugar isomerization by Sn‐Beta zeolite.

Preliminary experiments employing a hydrothermally synthesized Sn‐Beta zeolite in HF medium (Sn‐Beta‐HF, Si/Sn=108) and a nanosized post‐synthetically modified catalyst (Sn‐Beta‐ps, Si/Sn=80) confirm that there is no effect of the crystallite size on the catalytic performance (Figure 1). From this, we conclude that sugar isomerization under these relatively mild conditions is not limited by internal mass transport limitations, as was shown before for the isomerization of arabinose.^26^

**Figure 1:**
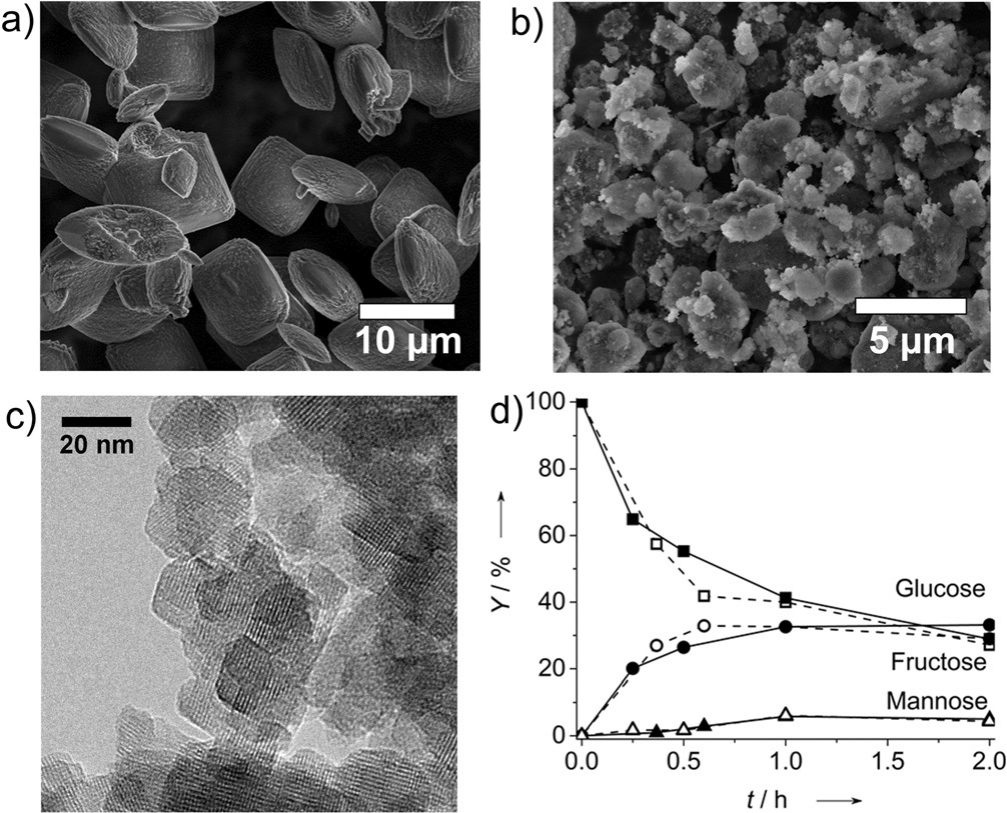
(a) SEM images of Sn‐Beta‐HF (b) SEM and (c) TEM images of nanosized Sn‐Beta‐ps obtained by post‐synthetic Sn modification. (d) Kinetic traces of glucose isomerization (open symbols/dashed line Sn‐Beta‐ps, closed symbols/solid lines Sn‐Beta‐HF).

Having excluded internal mass transport limitations, we focused on the intrinsic reactivity of Sn‐Beta‐HF by studying the transformation of carbohydrate substrates using in situ NMR spectroscopy in detail. This was done by following the chemical nature of the carbohydrate species adsorbed in the Sn‐Beta‐HF zeolite catalyst in time. We first investigated room temperature sugar isomerization over Sn‐Beta‐HF dehydrated at 170 °C in vacuo for 3 h. The dehydrated Sn‐Beta‐HF sample was then impregnated with a concentrated solution of DHA in D_2_O (DHA/Sn=2), followed by brief evacuation to remove bulk D_2_O. The role of the interaction of the sugars with Lewis acid sites in Sn‐Beta during sugar uptake has already been demonstrated.^27^, ^28^

Relevant ^13^C NMR spectra (Figure S1 in the Supporting Information) prove that DHA was converted into lactic acid (LA), as evident from the decrease of the DHA dimer signal (*δ*=65 ppm) and the appearance of signals related to LA at *δ*=20 ppm for −CH_3_, at *δ*=67 ppm for C−OH and at *δ*=179 ppm C=O. We confirmed that DHA isomerization did not occur over Al‐Beta and dealuminated Beta under the given conditions. In an experiment where the amount of substrate was increased to DHA/Sn=10, we found that the reaction went to completion in about 10 h (Figure 2). In this case, we observed an additional signal at *δ*=205 ppm owing to pyruvaldehyde, a known intermediate in the reaction from DHA to LA.^29^ We also evaluated the performance of Sn‐MCM‐41 at room temperature (Figure S2) and confirmed that its intrinsic reaction rate is significantly lower than that of Sn‐Beta‐HF in line with recently reported results.^30^ The sharper lines with Sn‐MCM‐41 as the catalyst are a result of the high mobility of the substrate molecules inside the large MCM‐41 mesopores. Owing to the severe overlap of signals from the substrate and products, only a qualitative assessment of the DHA conversion can be given on the basis of these NMR results.

**Figure 2:**
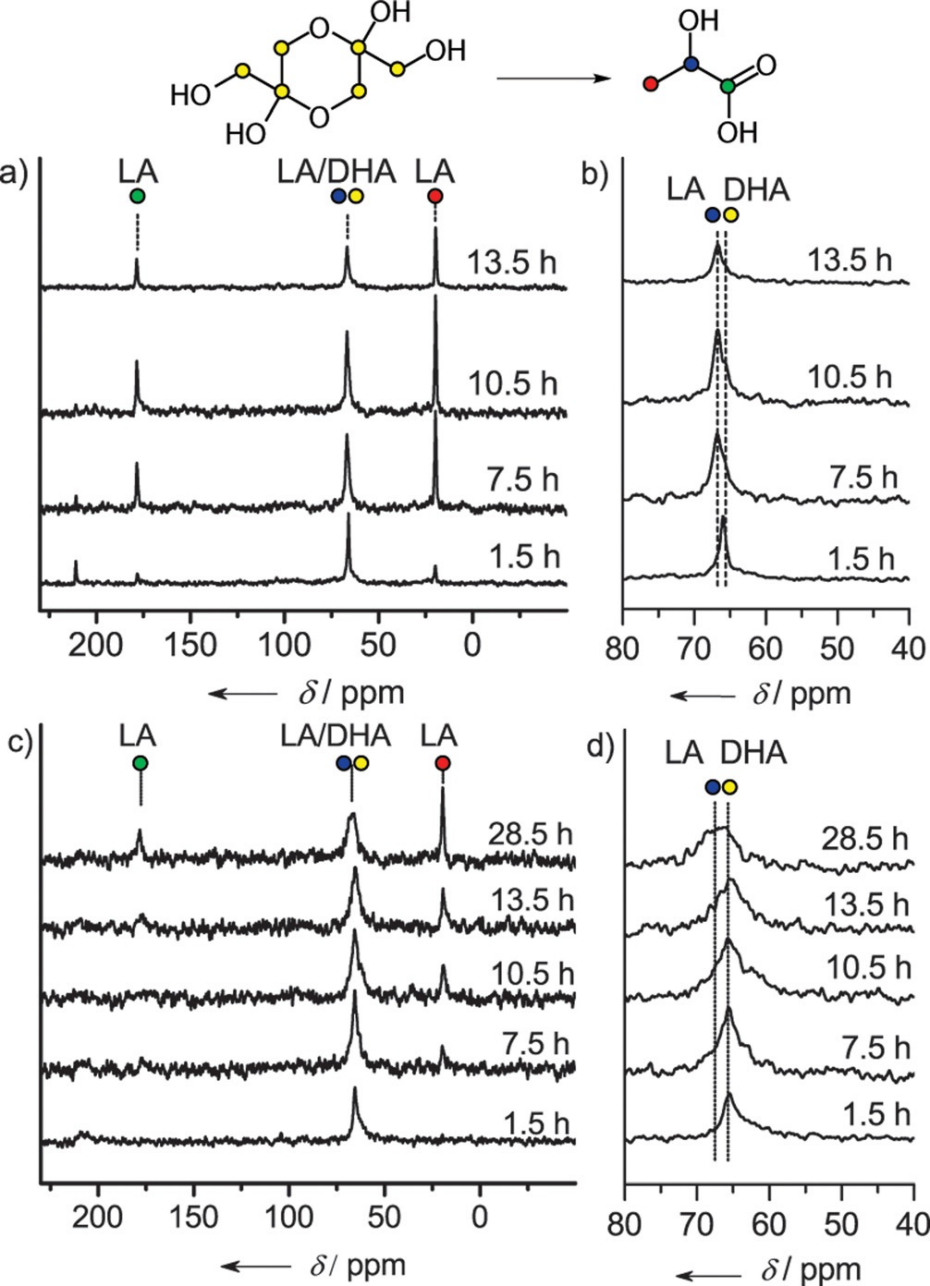
^13^C MAS NMR spectra of dehydrated (a, b) and hydrated (c, d) Sn‐Beta‐HF impregnated with a DHA/D_2_O solution (in situ NMR spectra recorded at room temperature; DHA/Sn=10).

As isomerization of sugars is typically carried out in water, we investigated the influence of pre‐adsorbed water on the isomerization reaction, For this purpose, we followed the conversion of DHA by ^13^C NMR spectroscopy of the same Sn‐Beta‐HF sample after hydration in saturated water vapor (50 °C, 12.3 kPa). Thermogravimetric analysis showed that this hydration procedure led to a water content of Sn‐Beta‐HF of 2.4 wt %. When this zeolite was then impregnated with the same DHA/D_2_O solution (DHA/Sn=10) as used before for the dehydrated zeolite, we observed that the conversion of DHA proceeded much slower. After approximately 30 h, the conversion was not complete. This experiment shows that the presence of water molecules in the micropores of Sn‐Beta‐HF significantly slows the reaction of DHA under these conditions.

Similarly, we investigated the conversion of d‐glucose in Sn‐Beta‐HF. In this case, we used direct excitation ^13^C magnetic angle spinning (MAS)NMR allowing for quantitative monitoring of sugar conversion. Introducing a ^13^C label in the C1 position of d‐glucose helped to quantify reactant and product. First, the reactivity of dehydrated Sn‐Beta‐HF was assessed by room‐temperature impregnation of a d‐glucose/D_2_O mixture (d‐glucose/Sn=5). d‐fructose was one of the reaction products, as follows from the appearance of new signals in the ^13^C NMR spectra at *δ*=62 and 64 ppm, which respectively relate to β‐furanose and β‐pyranose forms of d‐fructose.^31^ At the same time, the signals at *δ*=96 and 92 ppm, related to the α‐ and β‐anomers of d‐glucose, (Figure 3 a) decreased. Based on the ^13^C intensities of the reactant and products, we estimate that about 50 % of the starting d‐glucose was converted in 12 h, mostly into d‐fructose (33 %). This approaches the glucose/fructose equilibrium ratio of 1.13 at room temperature.^8^, ^32^ The remainder of the ^13^C labels were found in the carbonyl region (170–180 ppm), which should be related to secondary reaction products such as 5‐hydroxymethylfurfural.^33^ The impregnation of hydrated Sn‐Beta‐HF with the same solution resulted in a significantly lower reaction (Figure 3 b), in keeping with the difference observed for DHA isomerization.

**Figure 3:**
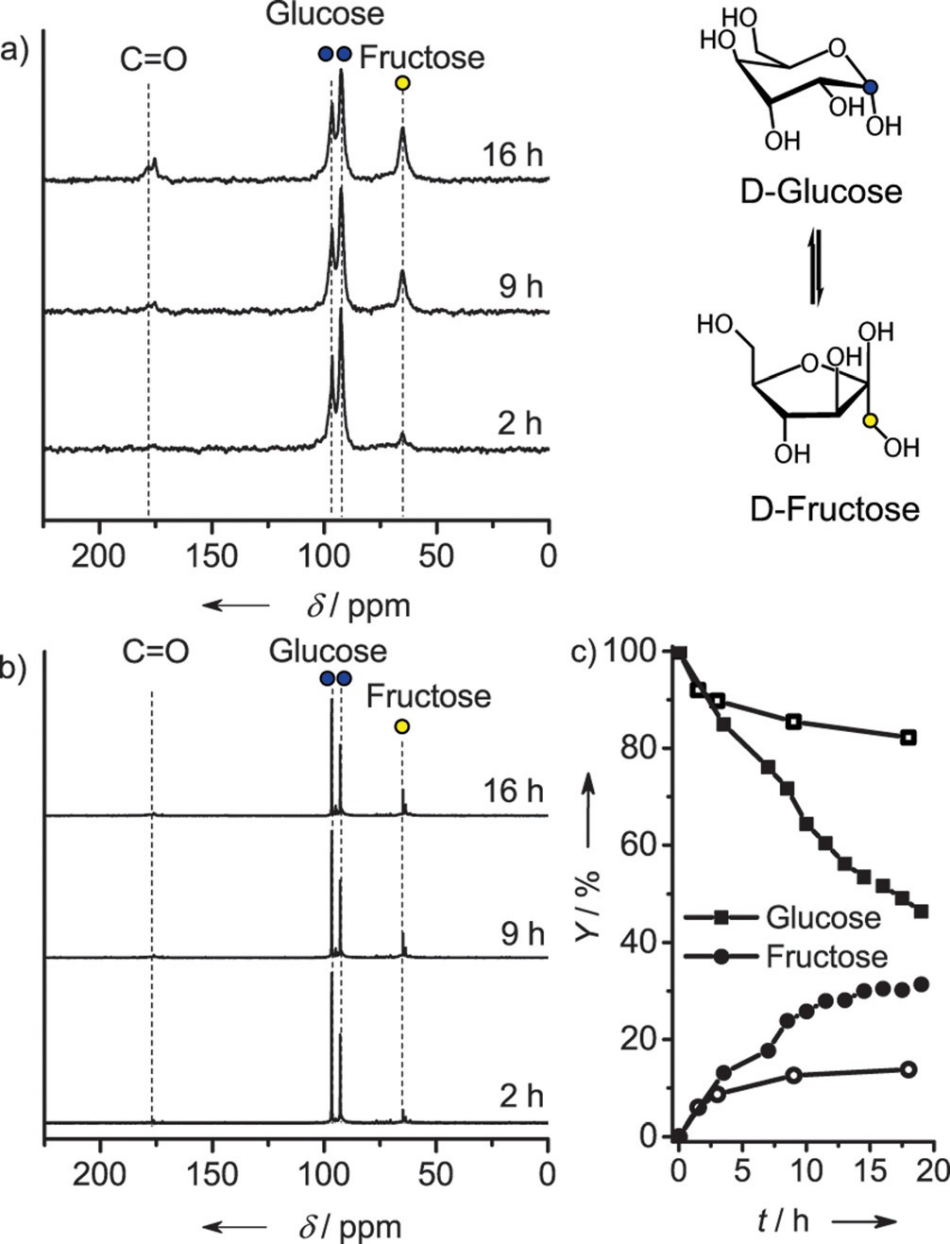
^13^C MAS NMR spectra of dehydrated (a) and hydrated (b) Sn‐Beta‐HF impregnated with a ^13^C1‐d‐glucose/D_2_O solution and (c) the conversion profile derived from quantification of the spectra. Glucose and fructose concentrations are indicated for hydrated (open symbols) and dehydrated (closed symbols) Sn‐Beta‐HF (in situ NMR spectra recorded at room temperature).

In these experiments, we observed that the concentration of the α‐anomer of d‐glucose was higher than that of the β‐anomer. This could be attributed to a confinement effect. The increased concentration of one of the anomers in the zeolite micropores should not affect the catalytic performance, as the barrier for mutarotation on Sn sites is much lower than the barrier for the H‐shift reaction.^34^, ^35^

Figure 3 c summarizes the sugar yields calculated from the NMR. One can see that for the hydrated Sn‐Beta‐HF, the reaction proceeds very slowly after 4 h and the conversion stays below 20 % even at prolonged reaction times. On contrary, dehydrated Sn‐Beta‐HF afforded a glucose conversion of about 50 % after 12 h. The data show that cyclic d‐fructose was formed much faster than previously reported in a similar NMR experiment at room temperature.^33^

Apart from signals corresponding to d‐fructose, the ^13^C NMR spectra also contained signals in the carbonyl region (*δ*=160–200 ppm). One of the signals (*δ*=178.6 ppm) is related to 5‐hydroxymethylfurfural.^33^ The other signals could not be identified. They are not resulting from the acyclic aldohexoses, because these compounds would give rise to a signal at *δ*=205 ppm.^36^

After 22 h of room temperature reaction, the rotor and its contents were placed in an oven at 100 °C for 2 h. ^13^C NMR spectra recorded after cooling to room temperature evidenced that glucose and fructose were completely converted as followed by the absence of signals in the 60–100 ppm range (Figure S3). This is surprising, as in batch experiments the isomerization reaction goes to equilibrium (glucose/fructose≈0.75 at 100 °C)^8^, ^32^ with comparatively little formation of byproducts.^8^ Instead, the spectrum of the heated sample contained signals owing to carbonyl groups (*δ*≈180 ppm) and CH_3_ groups (*δ*≈20 ppm). These findings show that the sugars were also converted to lactic acid. We postulate a reaction mechanism (Scheme S1) that helps to explain the occurrence of ^13^C labels at −CH_3_ and carbonyl carbon atoms following reactions of ^13^C1‐d‐glucose. This mechanism is consistent with recent insights from Lewis acid‐catalyzed retro‐aldolization of hexoses.^13^, ^37^

In the light of the presented data, we hypothesize that the sugar reactant and water solvent molecules compete for coordination to Sn sites. In this way, differences in the coverage of the active sites with sugar would affect the catalytic activity. Previously a similar hypothesis has been put forward to rationalize the crucial role of hydrophobic reaction environment on glucose isomerization reaction.^38^ This taken together with our findings inspired us to further explore whether competitive adsorption effects play a role during sugar isomerization by comparing catalytic performance of Sn‐Beta‐HF in water, in ethanol/water=9/1 *v*/*v* and in THF/water=4/1 *v*/*v* mixtures (THF=tetrahydrofuran). The solvent mixtures were selected to ensure sufficient variation in the adsorption strength with Sn sites as compared with water, yet to still be able to dissolve the sugar reactant. Figure 4 summarizes the results of these activity measurements at 90 °C. In the THF/water mixture the isomerization activity is much lower than the reference case in water. On the other hand, the reaction rate was much higher when the reaction was carried out in the mixed ethanol/water solvent.

**Figure 4:**
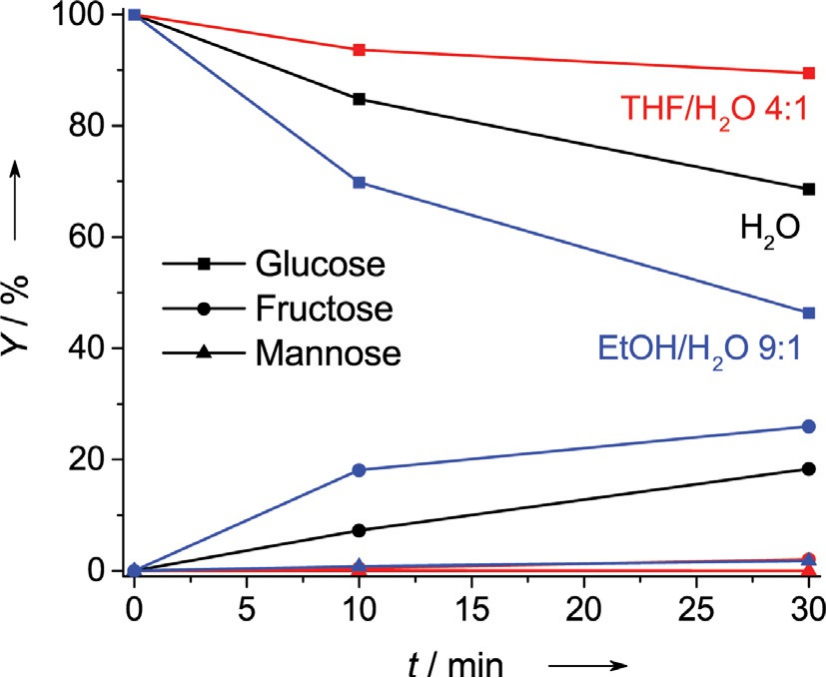
Batch isomerization of glucose over Sn‐Beta‐HF in THF/water 4:1 *v*/*v* (red), water (black), and EtOH/water 9:1 *v*/*v* (blue); reaction conditions: 90 °C, 40 mg catalyst, 2.5 mL 125 mm glucose solution.

Uptake measurements of the different solvents on Sn‐Beta‐HF (Figure S6) show that the adsorption of THF and ethanol on the zeolite is much stronger than that of water at room temperature and also at 90 °C. This indicates that the interaction differences with zeolite Beta itself cannot explain the activity differences, but should rather be related to the differences in specific interactions with the active Sn‐sites, that is, competitive adsorption. Further support for the hypothesis that competitive adsorption between the solvent and sugars affects catalytic performance was obtained from periodic density functional theory (DFT) calculations (Figure 5). For this purpose, we used a complete zeolite Beta unit cell containing an open lattice Sn site (Sn/Si=63). This choice was based on recent studies emphasizing the role of such partially hydrolyzed open lattice sites as the active centers for sugar isomerization in Sn‐Beta zeolite catalysts.^31^, ^33^, ^34^

**Figure 5:**
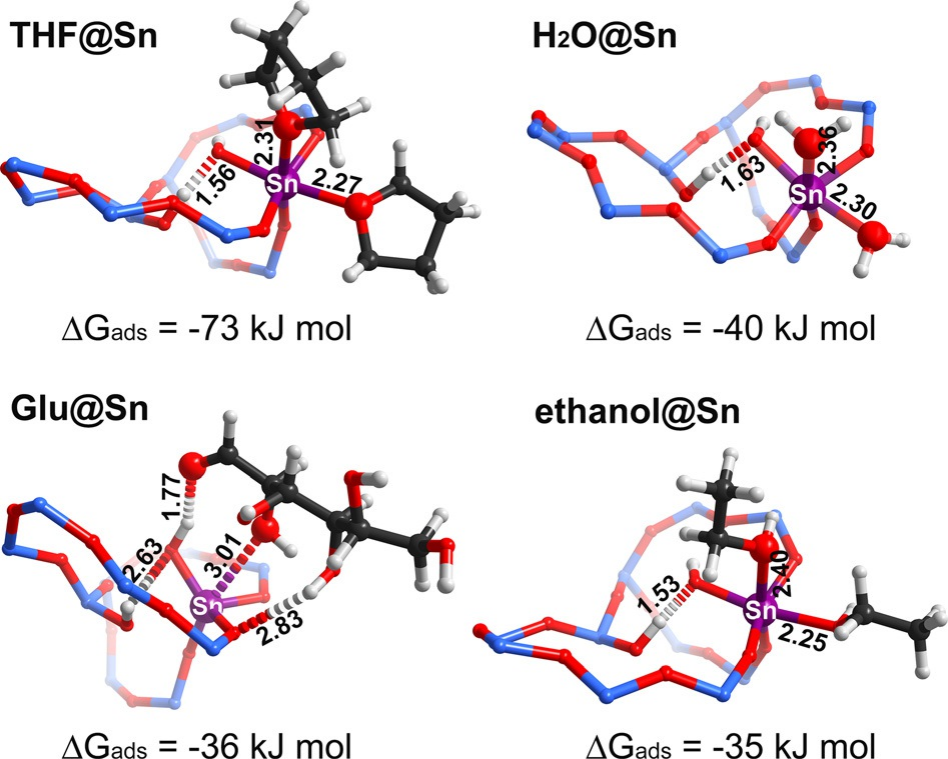
Structures and adsorption energies (Δ*G*_ads_) of two molecules of adsorbed water, THF, and ethanol and one molecule of glucose (Glu) on the open Sn site in Sn‐Beta. For clarity, only part of the periodic zeolite model is shown (bond lengths in Å).

The adsorption configurations were constructed following the results of a previous theoretical study on the mechanism of glucose isomerization by Sn‐Beta.^39^ We determined the specific interaction energies of adsorbates with the Sn sites (Table S3, Figure S5) and neglected van der Waals (vdW) interactions with the zeolite pore wall, as the dispersion‐corrected DFT (DFT‐D) method tends to overestimate the vdW contribution.^40^ An open Sn site can favorably coordinate two water molecules, two THF and two ethanol molecules or one acyclic glucose molecule. Ethanol adsorbs much weaker on the open Sn site (−43 kJ mol^−1^) than THF, water, and acyclic glucose (−100, −58, and −48 kJ mol^−1^, respectively; Table S3). Here we chose acyclic glucose as the reference state over glucopyranose dominating the solution because the interaction of the former with the catalytic Sn sites is much stronger and it is also required to enable the rate‐determining H‐shift reaction within the adsorption complex (Table S3). The current findings on the preferred adsorption of acyclic glucose over glucopyranose on open Sn site are in an apparent disagreement with the previous mechanistic studies,^33^, ^35^, ^41^ in which a metastable semi‐open configuration of the acyclic glucose has been discussed as a reaction intermediate within the catalytic path from glucose to fructose. In this work, we selected the most stable conformation of the acyclic glucose inside beta micropores characterized by a fully stretched and open carbohydrate backbone. The very strong adsorption of THF to the active Sn centers hinders the adsorption of glucose. In other words, the replacement of THF by glucose is endothermic, which will lead to low coverage with the sugar reactant during isomerization catalysis. On the other hand, ethanol can be easily displaced by glucose and this will increase the coverage and activity. The influence of temperature and entropy on the adsorption process over the open site is further evaluated by calculation of the Gibbs free energy of adsorption at a typical temperature of 298 K and 1 atm. The data in Figure 5 show that the entropy losses owing to adsorption in the pores substantially decrease the favorability of the confinement of all substances inside the zeolite as compared to the energetics predicted on the basis of the electronic DFT energies. However, the qualitative trends in the interaction strengths are not affected by correcting for the entropic effects. The differences in isomerization activity can thus be understood in terms of steady‐state coverage of Sn centers with the sugar reactant. The stronger the interaction with the solvent molecules, the lower the coverage and the isomerization activity are. The increased activity seen for the dehydrated zeolite in the in situ NMR experiment can be explained by the lower coverage of Sn centers with water as compared with the hydrated zeolite.

Qualitatively similar results have been obtained with an alternative semi‐open Sn(μ‐OH)_2_Si configuration identified as intrinsically more stable site in the current periodic Sn‐Beta model (see the Supporting Information). Because of the 5‐fold coordination of the Sn center in the semi‐open configuration, it can only accommodate one solvent molecule in the first coordination shell of Sn (Figure S5). DFT calculations predict very similar adsorption energies for water, ethanol, and acyclic glucose on semi‐open SnOH site in zeolite Beta (−49, −56, and −47 kJ mol^−1^, respectively). Similar to the true open SnOH, this site forms very strong complexes with THF (−75 kJ mol^−1^) and binds glucopyranose very weakly (−1 kJ mol^−1^).

In summary, we have shown that competitive adsorption between sugar molecules and polar solvent molecules for the Lewis acid Sn sites in Sn‐Beta affect the overall isomerization activity. Room‐temperature NMR experiments demonstrate that under solvent‐lean conditions trioses (DHA) and hexoses (glucose) are more rapidly isomerized than in the presence of water. DFT calculations provide further support for the hypothesis that water competes with these sugars for adsorption on open Sn sites embedded in the zeolite framework. A solvent, such as ethanol, that interacts weakly with the active Sn centers allows for higher isomerization activity; isomerization will be suppressed by a strongly interacting solvent, such as THF. The present results suggest the importance of competitive adsorption between the reactant and polar solvent molecules during sugar conversion reactions in Sn‐Beta isomerization catalysts. These insights help to identify improved reaction conditions for the efficient isomerization of glucose, which is an essential step in the valorization of lignocellulosic biomass.

## Supporting information

As a service to our authors and readers, this journal provides supporting information supplied by the authors. Such materials are peer reviewed and may be re‐organized for online delivery, but are not copy‐edited or typeset. Technical support issues arising from supporting information (other than missing files) should be addressed to the authors.

Supplementary
