# Supplementary material for: Competitive Adsorption of Substrate and Solvent in Sn‐Beta Zeolite During Sugar Isomerization
Source: ChemSusChem. 2016 Oct 28;9(22):3145–9. doi: 10.1002/cssc.201600800 (PMC5132075; doi:10.1002/cssc.201600800)
Supplement: Supplementary file 1 — Supplementary [file CSSC-9-3145-s001.pdf]

CHEMISTRY & SUSTAINABILITY
CHEMSUSCHEM
ENERGY & MATERIALS

Supporting Information

Competitive Adsorption of Substrate and Solvent in Sn-Beta Zeolite During Sugar Isomerization

William N. P. van der Graaff,[a] Christiaan H. L. Tempelman,[a] Guanna Li,[a] Brahim Mezari,[a] Nikolay Kosinov,[a] Evgeny A. Pidko,*[a, b, c] and Emiel J. M. Hensen*[a]

cssc_201600800_sm_miscellaneous_information.pdf

Supplementary information

1. Experimental

1.1. Chemicals

Tetraethylammonium hydroxide (TEAOH, 35 wt% in H_2O, Aldrich), anhydrous $SnCl_4$ (99%, Aldrich), Tetraethylorthosilicate (TEOS, synthesis grade, Merck), HF (40% in H_2O, Merck), $SnCl_4.5H_2O$ (98%, Acros), Cetyltrimethylammonium bromide (CTAB, > 98% Aldrich), Tetramethylammonium hydroxide (TMAOH, 25% in water, Aldrich), Beta zeolite (Si/Al 12, Akzo Nobel), HNO_3 (65 wt. % in water, Merck), Amberlyte-IRN78 (Supelco), 1,3-dihydroxyacetone (DHA, dimeric form, 97%, Aldrich), $^{13}C_1$-glucose (Cambrige Isotopes, 99%) were used as received without further purification.

1.2 Synthesis of Sn-Beta

Tetraethylorthosilicate (TEOS) (14.03g, 1 eq.), TEAOH (15.42g 35 wt. % solution, 0.54 eq.) and $SnCl_4.5H_2O$ (0.24g, 0.01 eq.) were mixed in a Teflon beaker until a clear, homogeneous gel was obtained. After evaporation of ethanol and part of the water, HF (40% in water, 1.5 mL, 0.50 eq.) was added to yield a dry gel with a final composition of 1 SiO_2: 0.54 TEAOH: 0.50 HF: 0.01 $SnCl_4$: 7.5 H_2O. The mixture was transferred to a Teflon-lined steel autoclave and heated in static mode for 40 days at 140 °C. Subsequently, the material was washed and dried in air. Calcination at 550 °C for 10h (1 °C/min) yielded the final material.

1.3 Synthesis of Sn-MCM-41

Prior to the synthesis of Sn-MCM-41, CTAB was ion-exchanged over a column containing Amberlyte-IRN78® (OH-form) to obtain the hydroxide form. The solution of cetyltrimethylammonium hydroxide (6 wt. % in water, 70.9 g) was mixed with $SnCl_4.5H_2O$ (1.25 g) and TMAOH (8.48g solution) in a Teflon beaker. Then, fumed SiO_2 (5.3 g) was added and the mixture was homogenized. Subsequently, the content was transferred to a Teflon-lined steel autoclave and heated at 135 °C for two days. The resulting material was washed with water and ethanol and dried in air. Calcination at 550 °C (10h, 1°C/min) yielded the final material.

1.4 Synthesis of Dealuminated Beta

Beta zeolite (H-form) was dealuminated with 65wt. % HNO_3 (50 mL/g material) at 110 °C for 16h. After cooling down, the contents were filtered and washed until the filtrate was neutral. The material was obtained by drying in air.

1.5. Synthesis of postsynthetically synthesized Sn-Beta (Sn-Beta-ps)

Dealuminated Beta zeolite was dried in vacuo at 170°C for 3 h in a Schlenk flask. Sn was incorporated by adding an excess of anhydrous $SnCl_4$ at 100°C under an inert atmosphere and the mixture was

stirred overnight. In order to remove unreacted $SnCl_4$ from the zeolite pores, the materials were thoroughly washed with methanol at least six times and dried in air. The final material was obtained by calcination at 550 °C (1°C min^{-1}, 5 h).

2. General procedures

2.1 Impregnation procedure

The materials were impregnated with a solution of either DHA or $^{13}C_1$-glucose in D_2O. Typically, 0.12 mL of the substrate solution was thoroughly mixed with 0.12 g material (0.24 mL to 0.12 g in the case of Sn-MCM-41 with Substrate:Sn = 10). After short evacuation (~ 15 minutes), the contents were immediately transferred to a 4 mm zirconia rotor, after which the NMR measurements were started.

2.2 Characterization

X-ray diffraction patterns of the zeolite samples were recorded on a Bruker D4 Endeavor diffractometer using Cu Kα radiation in the 2θ range 5–60 ° with a scanning speed of 0.01 ° s^{-1}. The XRD pattern of Sn-MCM-41 was recorded in the 2θ range of 0.5 ° and 5 ° at a scanning speed of 0.0049 ° s^{-1}.

Elemental analyses were carried out using a Spectro Ciros CCD ICP optical emission spectrometer with axial plasma viewing. For analysis the materials $HF:HNO_3:H_2O$ 1:1:1 was used as matrix.

Thermogravimetric analyses to determine the water content of the solid samples were done on a Mettler TGA/DSC-1 apparatus using 70 µL alumina crucibles. Helium was used at a gas flow rate of 40 mL min^{-1}.

Argon physisorption measurements were performed at –186 °C on a Micromeritics ASAP2020 apparatus in static measurement mode. In a typical experiment, a zeolite sample (typically 100 mg) was outgassed at 200 °C for 8 h prior to the measurement. The N_2 sorption isotherms for Sn-MCM-41 were measured at −196 °C on a Micromeritics ASAP3020 Tristar system in static measurement mode. The sample was pretreated at 150 °C for several hours prior to the measurement. The Brunauer–Emmett–Teller (BET) equation was used to calculate the specific surface area from the adsorption data obtained. The mesopore volume and mesopore size distribution were calculated using the Barrett–Joyner–Halenda (BJH) method using the adsorption branch of the isotherm (2–50 nm).

^{13}C MAS NMR spectra were recorded using a Bruker DMX-500 NMR spectrometer using a 4 mm zirconia rotor at a spinning rate of 10 kHz at a frequency of 125 MHz. ^{13}C chemical shifts were referenced to adamantane. For Sn-Beta/DHA experiments, a relaxation delay time (D1) of 5 seconds was used and all spectra recorded were averaged over 1024 scans. For Sn-Beta/Glucose experiments D1 times of 20s and 40s were used and averaged over 256 and 128 scans, respectively. In the case of glucose, the D1 values were adjusted to enable quantitative analysis. Quantification of the signals was performed using the Fityk curve fitting program.

Transmission Electron Microcsopy (TEM) micrographs were acquired on a FEI Tecnai 20 transmission electron microscope (FEI company) at an acceleration voltage of 200 kV with a LaB6 filament. TEM sample preparation involved typically, sonication of the samples in pure ethanol and applying a few droplets of the suspension to a 200 mesh Cu TEM grid with a holey carbon support film. TEM images were recorded at different magnifications using a Gatan 1k x 1k CCD camera. Scanning electron microscopy (SEM) was performed using a Philips environmental FEIXL-30 ESEM FEG in high-vacuum mode at low voltage.

2.3. Batch sugar isomerization reactions

Batch sugar isomerization reactions were typically carried out by mixing 40 mg catalyst and 2.5 mL of a 125 mM glucose solution in a 4 mL thick-walled reaction vial. The reactions were carried out at 90°C and 100°C. The reaction mixture was analyzed by a Shimadzu HPLC using a Prevail Carbohydrate column (Grace, 4.6 x 250 mm, 5 (Grace, 4.6 x 250 mm column, 5 µm particle size) coupled to a Shimadzu ELSD-II detector. Analysis conditions were MeCN:H_2O 65:35, 0.75 mL/min, T = room temperature. ELSD settings were 50°C and 3.50 bar N_2.

3. Physicochemical properties of the synthesized materials

Table S1: Physicochemical properties of the synthesized materials.

Material	Si/Sn	Si/Al	V_{meso} ($cm^3\ g^{-1}$)	V_{micro} ($cm^3\ g^{-1}$)	S_{BET} ($m^2\ g^{-1}$)
Sn-Beta-HF[a)]	108	>1500	0.04 [a)]	0.21 [a)]	530 [a)]
Sn-MCM-41 [b)]	26	>1500	0.87 [b)]	< 0.01 [b)]	953 [b)]
Sn-Beta-ps	80	635	0.36	0.14	518
Al-Beta	-	12	0.29	0.15	479
Al-Beta-deal	-	635	0.39	0.17	530

a) Ar physisorption
b) N_2 physisorption

4. TGA of Sn-Beta-hydrated

Table S2: Water content of hydrated Sn-Beta as determined by TGA

Material	Sample weight (mg)	Weight loss (mg)	Water content (wt. %)*
Hydrated Sn-Beta-HF	9.45	0.22	2.40
Sn-Beta-HF	12.61	0.16	1.38
Sn-Beta-HF	16.65	0.22	1.32**

*Weight loss between 25 and 550°C

** Weight loss after 3 h 170°C.

5. Supplementary NMR spectra

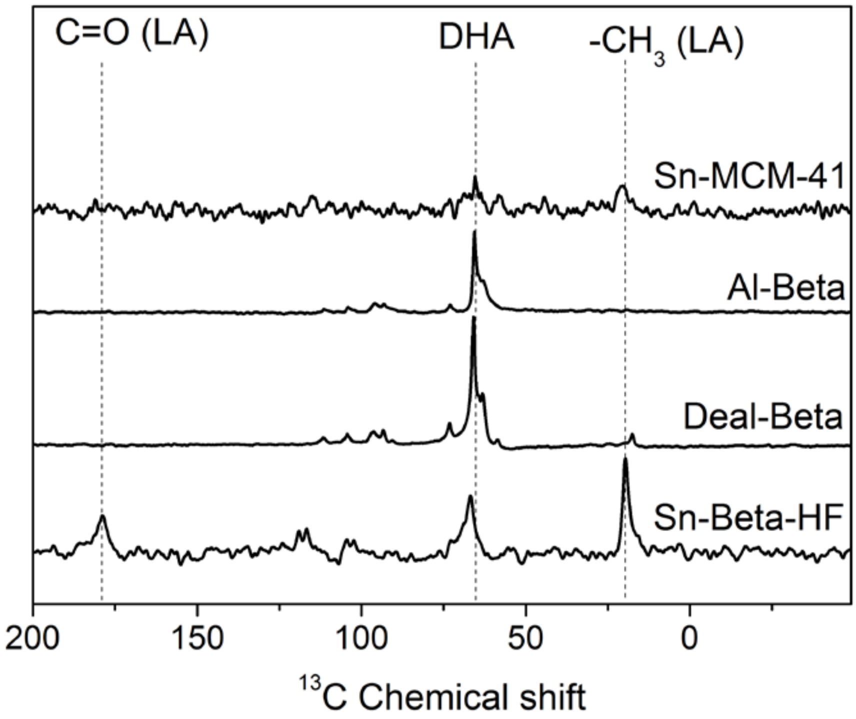

Figure S1: ^{13}C MAS NMR spectra of selected materials impregnated by DHA/D_2O. DHA: metal = 2, T = RT, t = 10.5h.

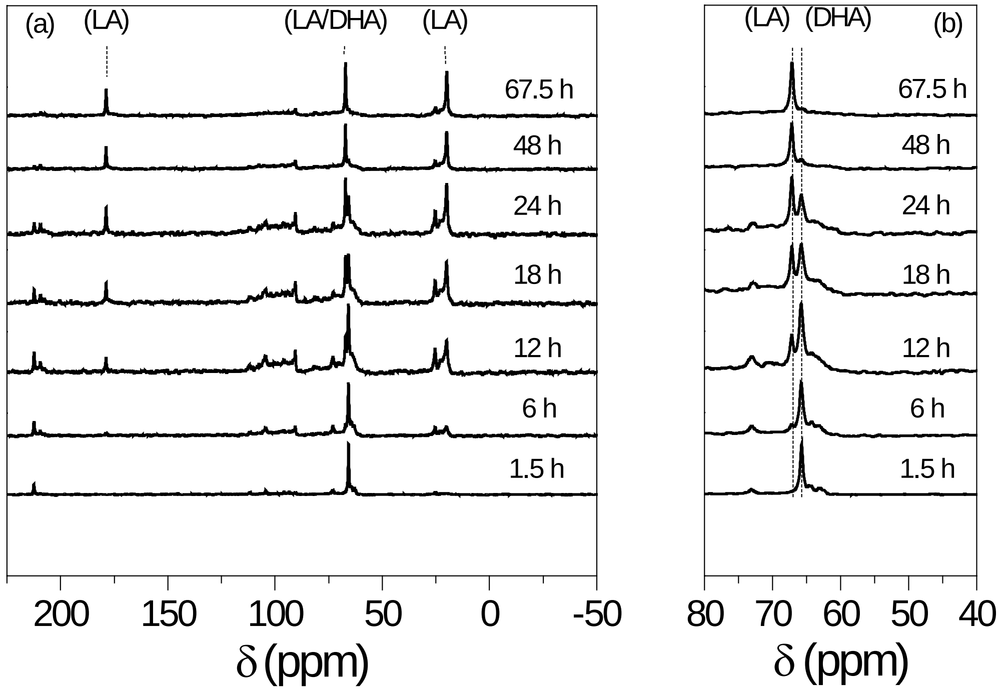

Figure S2: ^{13}C MAS NMR spectra representing materials after impregnating Sn-MCM-41 with a DHA/D_2O solution (DHA:Sn = 10) showing ranges of (a) the complete spectra and (b) zoomed-in.

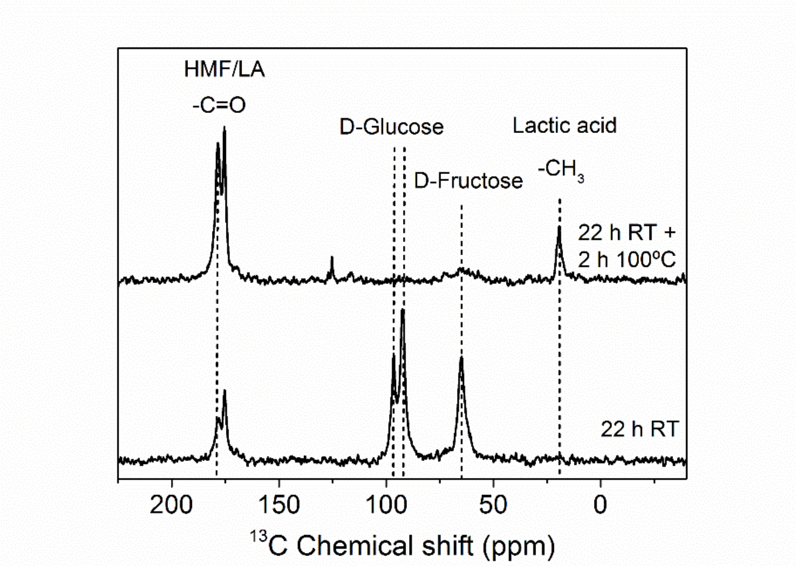

Figure S3: ^{13}C MAS NMR spectra of ^{13}C1-Glucose-impregnated dehydrated Sn-Beta-HF after 22 h reaction at room temperature and subsequent heating at 100°C for 2 h. 5-Hydroxymethylfurfural and lactic acid are indicated as HMF and LA, respectively.

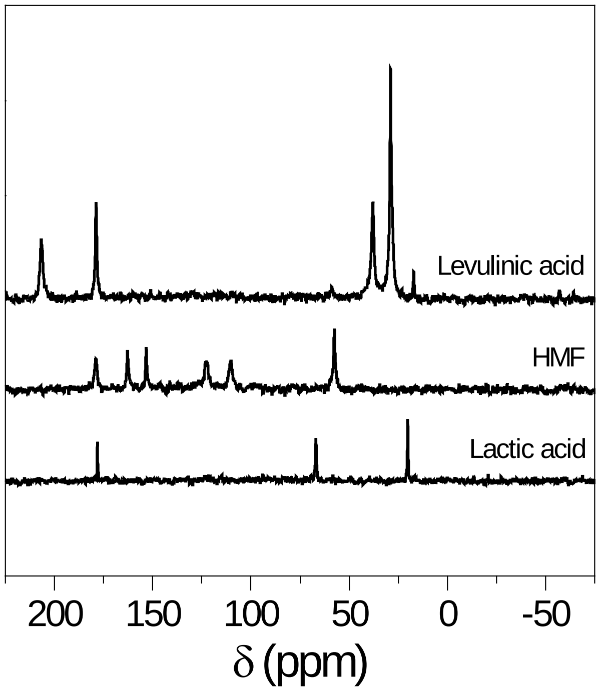

Figure S4: ^{13}C MAS NMR spectra representing Sn-Beta after chemical impregnation with levulinic acid, HMF and lactic acid.

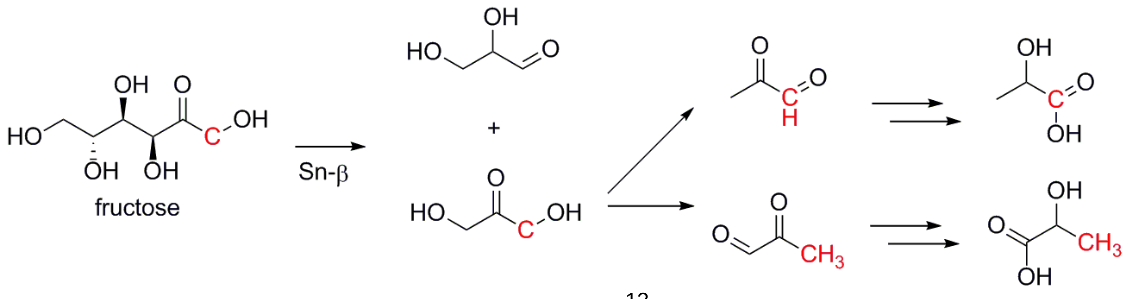

Scheme S1. Pathway explaining the occurrence of ^{13}C-labeled carbonyl and –CH_3-species.

6. Computational details

All DFT calculations were performed using the Vienna Ab initio Simulation Package (VASP 5.3.5) [1]. The exchange-correlation PBE functional based on generalized gradient approximation was employed [2]. The electron-ion interactions were described by the projected augmented wave (PAW) method [3]. Van der Waals interactions were accounted for by the DFT-D3 method. Geometry optimization was carried out with a plane-wave basis set with a cutoff energy of 400 eV. The calculations were assumed to be converged, when the forces on each atom were less than 0.05 eV/Å. Brillouin zone sampling was restricted to the Γ point. The periodic model of Beta polymorph A zeolite was used in this study [4]. Optimized cell parameters for all-silica zeolite Beta are a = b = 12.66 Å and c = 26.40 Å. We considered an open Sn-site and closed Sn-site as the active centers for sugar isomerization. The open site was generated by H_2O dissociation over a framework Sn-O-Si moiety. The framework tetrahedral Sn(IV) site ($Sn(O_FSi_F)_4$, O_F and Si_F represent the framework oxygen and silicon atoms, respectively) was generated by replacing a Si atom by a Sn atom at the T9 site of Beta. The thus obtained $(Si_FO_F)_3SnOH\cdots HO_FSi_F$ open Sn site can stabilize two solvent molecules with totally a octahedral geometry. For glucose, only one molecule can be stabilized on the Sn-site. In this work we focus on the analysis of specific interactions between the adsorbed molecules including both the substrate and the solvents with the Sn site. Although dispersion interactions were properly accounted for during geometry optimization, the analysis of the adsorption energies was carried out based on “specific adsorption energies” in which the van der Waals contribution was intentionally neglected. This is based on the consideration that the vdW correction is mainly caused by the non-bonding interactions between zeolite channels and adsorbates, but in this study we focus on the interaction strength of specific Sn Lewis acid and adsorbates which is dominanted by electrostatic energetics described by pure DFT part of the calculation. Therefore, only the pure PBE part of the interaction energy was analyzed as the dispersion interactions between the sugar and the zeolite walls are similar for the adsorption complexes formed at the reactive Sn site and for the physisorbed configurations at zeolite walls [5]. As it is shown in Table S3, over the closed Sn site, all solvents have stronger interaction with Sn site than that of sugar (entry 4, ΔE_{ads} of closed site), which indicates the lower reactivity of closed Sn site compared to open Sn site.

The original zeolite model used here was the most stable open SnOH site. This open configuration is not affected by the coordination of adsorbates to the Lewis acidic Sn site. DFT calcualtions identify an intrinsically more stable by 12 kJ/mol alternative configuration, in which the two terminal hydroxyl groups originally associated with a silanol and Sn-OH motiety occupy bridging (μ(OH)) positions between the lattice Si and Sn sites (semi-open configuration, Figure S5c). In view of the substantially different geometry of such a semi-open site, its reactivity towards glucose activation can be substantially different from that of the commonly-accepted open SnOH. Taking into account that there is no experimental evidence for the presence of such semi-open SnOH configurations in Sn-Beta

zeolites, the open SnOH model was selected for supporting computational studies on adsorption of the sugar substrate and solvents on Lewis acidic Sn sites.

Supplementary References:

1. G. Kresse and J. Hafner, *Phys. Rev. B*, **1993**, *48*, 13115-13118.
2. J. P. Perdew, K. Burke and M. Ernzerhof, *Phys. Rev. Lett.*, **1996**, *77*, 3865-3868.
3. P. E. Blöchl, Phys. Rev. B, 1994, 50, 17953-17979.
4. J.M. Newsam, M. M. J. Treacy, W. T. Koetsier; C. B. de Gruyter, *Proc. R. Soc. London, Ser. A*, **1988**, *420*, 375−405.
5. L. Yang, C. Adam, G. S. Nichol, S. L. Cockroft, *Nat. Chem.*, **2013**, *5*, 1006 – 1010.

7. Supplementary computational results

Table S3: Calculated adsorption energies of solvents, acyclic glucose and glucopyranose in the zeolites of Beta and Sn-BEA.

Adsorbates[a]	BEA[b] [d]ΔE_{ads}	BEA [e]ΔE^{DFTD3}_{ads}	Sn-BEA[c] Closed site ΔE_{ads}	Sn-BEA Closed site ΔE^{DFTD3}_{ads}	Sn-BEA Open site ΔE_{ads}	Sn-BEA Open site ΔG_{ads}[f]	Sn-BEA Open site ΔE^{DFTD3}_{ads}
Ethanol x2	-5	-111	-65	-176	-43	-35	-118
Water x2	-11	-42	-93	-131	-58	-40	-86
acyclic glucose x1	-20	-183	-43	-214	-48	-36	-218
THF x2	-24	-186	-87	-264	-100	-73	-274
Glucopyranose x1	-16	-156	-1	-209	0	6	-150

[a] Each Sn/Si site can accommodate two solvent molecules or one sugar molecule
[b] All-silica Beta
[c] One Sn atom at T9 site of BEA unit cell
[d] Adsorption energy without vdW correction; Energy is in the unit of kJ/mol
[e] Adsorption energy including vdW correction performed by DFT-D3 method
[f] Adsorption Gibbs free energy at 298 K, 1 atm.

Table S4: Relative stability of molecular acyclic glucose (o-Glu) and glucopyranose, and their adsorption energies in different BEA zeolite systems

	Gas Phase	ΔE_{ads} Si-BEA	ΔE_{ads} closed site/Sn-BEA	ΔE_{ads} open site/Sn-BEA
o-Glu	0	-20	-43	-48
Glucopyranose	-67	-16	-1	0

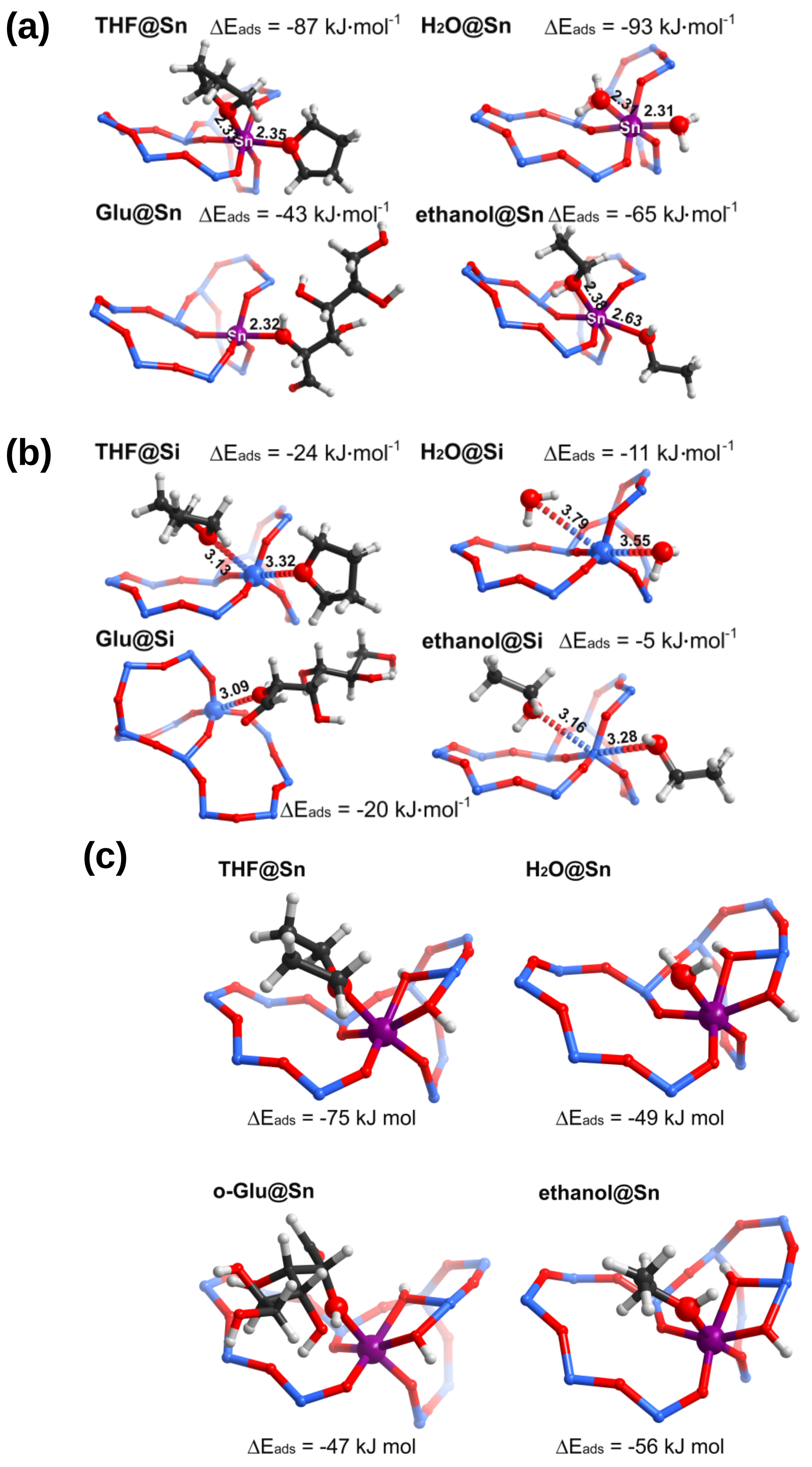

Figure S5: Local optimized structures and adsorption energies of two molecules of adsorbed water, THF and ethanol and one molecule of glucose (Glu) on the closed Sn site in Sn-Beta (a), on Si sites of the silicious walls of Beta (b), and one molecule on semi-open site (c). For clarity, only part of the periodic zeolite model is shown (bond lengths in Å).

8. Adsorption properties of Sn-Beta zeolite

Breakthrough adsorption experiments were conducted by leading either a THF-water (1 kPa – 1.7 kPa) or ethanol-water mixture (1 kPa – 1.7 kPa) in Ar (48 mL/min) over 750 mg of Sn-Beta-HF. Prior to measurement, the p_0 values of the individual components were determined. Quantification was performed by mass spectrometry (Balzers ThermoStar apparatus). The results show that the adsorption of THF is very strong compared to water. First, both water and THF are adsorbed, and after saturation, water is gradually forced out at the expense of water. Similar trends were observed for the ethanol/water mixture. Ethanol also adsorbs more strongly inside the pores than water – which suggests that the determining factor in the activity is the specific interaction of the solvent with the Sn-sites.

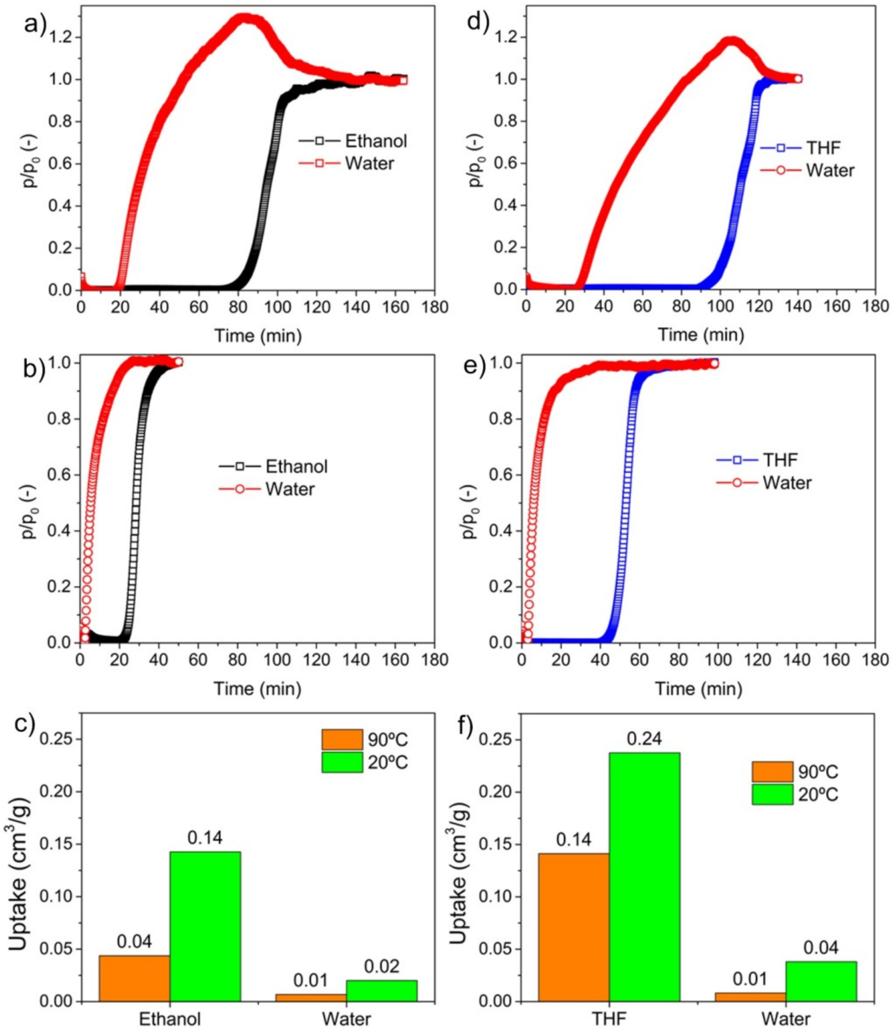

Figure S6: Breakthrough adsorption experiments of a-c) ethanol-water (1 kPa – 1.7 kPa) and d-f) THF-water (1 kPa – 1.7 kPa) at 20°C (a,d) and 90°C (b,d).

9. Coordinates of the optimized structures of both open and closed Sn-sites

Ethanol@open site

```
CONTCAR
   1.00000000000000
    12.6569995879999997    0.0000000000000000    0.0000000000000000
     0.0000000000000000   12.6569995879999997    0.0000000000000000
     0.0000000000000000    0.0000000000000000   26.3959999084000003
   O    Si   Sn   C    H
 131   63    1    4   14
Direct
  0.344271489  0.651407633  0.066253411
  0.349019047  0.814107505  0.001585074
  0.260586983  0.835271151  0.089558226
  0.468537542  0.818397943  0.082077782
  0.353103143  0.510840749  0.992087796
  0.235791553  0.473121201  0.073764697
  0.445879049  0.469406206  0.079359771
  0.709310376  0.661517614  0.064461448
  0.853284567  0.816336747  0.073045528
  0.665817835  0.843556155  0.109319646
  0.843877522  0.501613937  0.057659754
  0.653423188  0.470914929  0.095807645
  0.988207573  0.658166135  0.060516142
  0.051230269  0.845824528  0.094072855
  0.032002046  0.470155432  0.097191148
  0.716707372  0.315096018  0.551060560
  0.686301159  0.155769973  0.488344024
  0.764474536  0.122400857  0.580128531
  0.561400283  0.168645105  0.568329791
  0.727855014  0.473766695  0.483454339
  0.752205444  0.511787541  0.580432634
  0.530075382  0.610536407  0.603046665
  0.277097288  0.332276272  0.552967974
  0.160333890  0.163757428  0.564508521
  0.358852513  0.158850202  0.597315987
  0.145608576  0.498945965  0.545032522
  0.315245668  0.512831315  0.599310319
  0.028400029  0.332042497  0.563469578
  0.968385780  0.155822427  0.603169976
  0.962706110  0.525212430  0.592980533
  0.332669489  0.308328172  0.313570648
  0.182408532  0.324950946  0.243554341
  0.146794479  0.211916563  0.326656264
  0.156943001  0.421947123  0.329940053
  0.493763186  0.326716615  0.248621924
  0.515787755  0.216798156  0.333027553
  0.500577887  0.428010577  0.334457114
  0.342966524  0.701200325  0.316929735
  0.172238088  0.820758740  0.306785260
  0.156984345  0.630616122  0.348018452
  0.518012553  0.818373132  0.309092930
  0.526140774  0.632776266  0.352748692
  0.347661778  0.935138665  0.302451370
  0.182269175  0.008909227  0.351301607
  0.513309295  0.009267195  0.350959090
  0.686787964  0.657042258  0.833009444
  0.799852460  0.665572923  0.746952725
  0.856560737  0.779696890  0.827289794
  0.874884544  0.568701835  0.826386253
  0.564157445  0.756455643  0.771101673
  0.501561984  0.747011274  0.863649133
  0.502036612  0.565685115  0.819035562
  0.670369699  0.300445597  0.815572160
  0.835449159  0.169903271  0.812144004
  0.855083109  0.364078444  0.848518859
  0.514509451  0.161779376  0.828565944
  0.483017455  0.363425177  0.845458044
  0.676399605  0.030278645  0.812909076
  0.862537908  0.982577919  0.853212572
  0.493947964  0.957078090  0.845322144
  0.662275728  0.679789335  0.942818258
  0.694054568  0.835560830  0.008924605
  0.780864557  0.842441177  0.917176088
  0.574472949  0.874628793  0.931190711
  0.673504092  0.506924520  0.997652306
  0.783427235  0.514315085  0.913144741
```

0.573373795	0.501719625	0.909518382
0.298367058	0.673622812	0.931977929
0.171841630	0.836481020	0.945320228
0.363501672	0.869549604	0.907928686
0.179292002	0.501327985	0.935872719
0.366468240	0.498532172	0.891668636
0.047303548	0.665113940	0.937903292
0.987875492	0.848351124	0.898745614
0.988589011	0.480561784	0.898670645
0.362291591	0.327610207	0.440723049
0.324930238	0.160498437	0.499969094
0.260542224	0.161615875	0.406116503
0.464382791	0.146185246	0.424903704
0.328839618	0.499182129	0.498336472
0.279145786	0.502150325	0.401220549
0.478072893	0.500596757	0.428189273
0.717796474	0.312278145	0.420685254
0.855672986	0.155237065	0.427808428
0.661672518	0.123137136	0.391062920
0.864775215	0.463395886	0.408093216
0.667702248	0.501296298	0.386256475
0.000845429	0.312315673	0.432974556
0.054701458	0.119188369	0.407820989
0.070189625	0.504310495	0.412751782
0.658922695	0.368498125	0.689747021
0.825702218	0.334463076	0.749677312
0.810992443	0.229312692	0.663560497
0.848107170	0.436541174	0.663977129
0.509586001	0.293820686	0.750545883
0.497616760	0.264981379	0.652009285
0.463797854	0.455241063	0.690327635
0.647054044	0.713985232	0.681306036
0.821546791	0.834300766	0.688848006
0.831737775	0.643473606	0.647778510
0.502774077	0.866867144	0.688405045
0.438705097	0.675307167	0.704957167
0.674555069	0.985435623	0.693736089
0.862122746	0.025040742	0.655544101
0.499939663	0.055728536	0.649396788
0.345441149	0.660803194	0.182378986
0.192981367	0.644076364	0.250977847
0.158348890	0.752966223	0.168154191
0.173943202	0.544649094	0.163927786
0.494494528	0.641639488	0.253822679
0.526097596	0.765002044	0.175484642
0.522962295	0.555494166	0.163799688
0.344276300	0.285741240	0.181203168
0.180289831	0.154227438	0.185190400
0.158602281	0.341157157	0.144069260
0.512533138	0.157921472	0.188976520
0.533036202	0.347855653	0.150392650
0.348788380	0.028457472	0.193548829
0.170566550	0.960057279	0.153390898
0.525033410	0.967357160	0.149866574
0.993507293	0.823826565	0.998233836
0.012609889	0.498041018	0.997609508
0.994489967	0.163682762	0.503218197
0.963049876	0.472848081	0.496525976
0.181788401	0.997057398	0.251511578
0.517643840	0.990597982	0.250481281
0.842088501	0.995701962	0.754081534
0.509361032	0.030814228	0.750930004
0.561158576	0.454098821	0.544667952
0.347920054	0.738659359	0.613287275
0.259906404	0.572234044	0.695895681
0.356204746	0.778476886	0.060097194
0.344824332	0.525790877	0.053148042
0.730198137	0.788268545	0.063516189
0.720196355	0.535549223	0.053560661
0.971667479	0.785039552	0.056060219
0.967948536	0.532117902	0.053064000
0.545082485	0.848354524	0.129468571
0.680283635	0.191810470	0.547285577
0.686999714	0.439104286	0.539566302
0.280651041	0.204854678	0.553904410
0.268814011	0.459966035	0.549666572
0.038862687	0.204713944	0.557902263
0.025227065	0.457188190	0.549876535
0.480932159	0.163405022	0.616604660

0.205520041	0.316719465	0.303888223
0.460580653	0.320216854	0.307985358
0.216952637	0.699893898	0.305262959
0.469973157	0.698961360	0.307968257
0.220620537	0.939594296	0.303248135
0.475150729	0.938725397	0.303236131
0.166352338	0.513655960	0.372867352
0.804660842	0.668968381	0.808341622
0.562543467	0.679967619	0.820671344
0.796581193	0.291915348	0.806127941
0.544214140	0.280482569	0.809249494
0.803500545	0.046026744	0.807889465
0.549001635	0.044672374	0.809473503
0.873402187	0.481271552	0.871868678
0.677625370	0.806917249	0.949615046
0.673327272	0.551560427	0.939707084
0.294343227	0.797825126	0.946733205
0.299554696	0.546113051	0.938542875
0.050811529	0.792769319	0.945119479
0.056955034	0.537097328	0.942904044
0.484334869	0.864454283	0.887186195
0.353889035	0.199879133	0.443236878
0.361633276	0.455891775	0.442884942
0.731547032	0.187146312	0.432566192
0.743639301	0.437924972	0.425144041
0.975477504	0.188031722	0.443357987
0.974676398	0.438064267	0.437511080
0.537562441	0.124756431	0.374915185
0.784060037	0.341156022	0.691331865
0.531139758	0.349066777	0.695442919
0.775634594	0.714573647	0.691258090
0.538145130	0.751709340	0.710915713
0.799709443	0.959518164	0.698561857
0.546722681	0.987330528	0.695790709
0.847290810	0.528139670	0.620731447
0.218474394	0.650193634	0.190925156
0.472021793	0.654786239	0.193604728
0.217218836	0.276741842	0.188940603
0.470476034	0.279051484	0.192524567
0.221604630	0.035383996	0.195968216
0.476528087	0.035854478	0.196194543
0.150720935	0.458398520	0.119726369
0.159518398	0.846788594	0.126241238
0.538072442	0.461548618	0.122287931
0.850843959	0.136255848	0.625350576
0.159964728	0.127118135	0.372860212
0.542538242	0.514765035	0.375213234
0.870914683	0.862442639	0.874384899
0.482382161	0.483076220	0.866701766
0.408555336	0.574832975	0.648985396
0.372507967	0.760304500	0.559823028
0.169193370	0.637403732	0.680407448
0.338230396	0.870057520	0.544527497
0.078297666	0.623109774	0.716641494
0.592920381	0.630877057	0.622283894
0.543705260	0.519713306	0.565204968
0.382094711	0.793499042	0.633890572
0.330413123	0.698804687	0.538640199
0.457679439	0.748307433	0.554195800
0.150333036	0.609937998	0.642077502
0.194981579	0.720275138	0.678103115
0.285558901	0.597989716	0.728690731
0.252837935	0.881765412	0.549373556
0.357850386	0.882326420	0.504321954
0.380499968	0.931039052	0.566269388
0.051230740	0.540719129	0.717814002
0.012451380	0.672754724	0.703656979
0.098806980	0.648607549	0.755277721

H2O@open site

CONTCAR
1.0

12.6569995880	0.0000000000	0.0000000000
0.0000000000	12.6569995880	0.0000000000
0.0000000000	0.0000000000	26.3959999084

O	Si	Sn	H
131	63	1	6

Direct

0.340329915	0.653887808	0.067418933
0.343844086	0.816608846	0.003470052
0.265550226	0.841277659	0.094323620
0.470481873	0.816547871	0.081724323
0.352717787	0.513170421	0.992926359
0.236766383	0.473376423	0.074771658
0.446790785	0.473414600	0.079682149
0.707641423	0.662048459	0.064816035
0.853952289	0.814361632	0.073922642
0.665867805	0.845055044	0.109811485
0.844040275	0.503174007	0.058070511
0.653804123	0.471526682	0.096658751
0.989815474	0.657741129	0.059669260
0.054835025	0.842363894	0.093548045
0.032474015	0.470978141	0.097328573
0.726747334	0.311891109	0.551667809
0.687337399	0.148397893	0.492059946
0.766254842	0.119220071	0.583941519
0.565348268	0.175344422	0.572152674
0.718837023	0.476783574	0.487028927
0.753100514	0.509208262	0.583578229
0.511339009	0.620734394	0.592320800
0.267122686	0.326165468	0.546297133
0.160098940	0.156952187	0.564188838
0.359563977	0.163480714	0.596420765
0.148860380	0.499947160	0.547722459
0.317113340	0.479440689	0.606478393
0.033274002	0.330563515	0.564727545
0.971011460	0.154457599	0.604309559
0.964796722	0.522193968	0.594806015
0.333662212	0.304262817	0.315220624
0.184968308	0.329428703	0.245192811
0.144714519	0.213550940	0.327063292
0.161315158	0.422988325	0.332207024
0.492033958	0.327628195	0.248670623
0.520287097	0.218901768	0.332965672
0.497538328	0.429213434	0.334654510
0.343709230	0.700024128	0.317973197
0.172986746	0.819991171	0.307637691
0.158902973	0.630975425	0.349626899
0.513886750	0.820835054	0.309096575
0.527774930	0.633384883	0.352842689
0.343118429	0.941822588	0.304589242
0.170391068	0.008731945	0.351292044
0.513857782	0.011409744	0.350359797
0.684894562	0.661604822	0.833230793
0.795970559	0.665625811	0.747142017
0.857351422	0.778591871	0.827394605
0.870846033	0.567166090	0.826360345
0.558003068	0.748925865	0.767430305
0.500477076	0.751003742	0.861402750
0.502038658	0.565893114	0.819695532
0.670763612	0.300668836	0.816180408
0.835780382	0.168811947	0.812945843
0.853965402	0.363171101	0.850733697
0.513264000	0.165052116	0.826900959
0.483597279	0.364747345	0.846540093
0.675756514	0.031907406	0.813918412
0.860532999	0.981277406	0.854545891
0.493378192	0.962114930	0.847561598
0.660069287	0.679543972	0.944156349
0.695298731	0.836128116	0.009387969
0.781386554	0.838686824	0.917609811
0.575187862	0.873733640	0.931358755
0.673549652	0.506221592	0.998407483
0.784607589	0.516745985	0.914533257
0.574749172	0.501765370	0.910096347
0.298994660	0.675032556	0.932991982
0.171101153	0.837139428	0.945365429
0.364559323	0.870133162	0.909480870
0.179587007	0.502144098	0.935974777
0.367429316	0.500206053	0.892577767
0.047575157	0.665187001	0.939087987
0.987771928	0.847083449	0.898879826
0.988789558	0.482394755	0.898409009
0.361347765	0.327376932	0.442927718
0.324970573	0.153923988	0.498906225
0.258862913	0.161813095	0.405323297

0.463195413	0.148019418	0.423984349
0.337277412	0.498440325	0.503657639
0.269379705	0.501870453	0.408783048
0.472882211	0.502878428	0.428297788
0.706058741	0.310584784	0.427434117
0.853085935	0.160882801	0.429362535
0.660777032	0.122237183	0.393469095
0.857690692	0.456610739	0.412580788
0.663455486	0.498004854	0.388735175
0.003367937	0.313689798	0.434840143
0.051963456	0.121832527	0.408873886
0.059657585	0.507674634	0.410772651
0.663603485	0.360871375	0.688280463
0.827296495	0.334785879	0.751705825
0.819588482	0.226384938	0.666565657
0.852435708	0.435349643	0.666313410
0.510327458	0.304194599	0.750785291
0.488186628	0.275012672	0.652881205
0.482251912	0.470166773	0.690492153
0.642225683	0.701611221	0.678893387
0.804727554	0.834854305	0.688983917
0.834310472	0.642708421	0.649250984
0.518746972	0.871329427	0.686414242
0.428220838	0.688783467	0.695554256
0.678082705	0.005755908	0.694018185
0.869365990	0.021233780	0.657710969
0.497677803	0.064827196	0.652355433
0.345906526	0.662348330	0.182903126
0.194488078	0.643451571	0.252069175
0.160153806	0.758256972	0.171019658
0.173157513	0.549448609	0.163673595
0.495159507	0.642798483	0.254272431
0.526448965	0.766232610	0.175917253
0.523361087	0.556816161	0.164545178
0.342506081	0.288666457	0.180961028
0.178665355	0.157489941	0.188011363
0.156965166	0.344395190	0.145847693
0.506274283	0.157878995	0.188924536
0.531576455	0.349039227	0.150725111
0.342185229	0.025520496	0.193055123
0.157386020	0.966331005	0.153787449
0.520722389	0.967818260	0.149715573
0.992284894	0.824618220	0.998435736
0.012425942	0.496553242	0.997599721
0.991570890	0.164570227	0.504409432
0.965896070	0.472999603	0.498382181
0.174497396	0.995533407	0.251960278
0.511748850	0.991312742	0.250093371
0.841526091	0.991488993	0.755728900
0.509049118	0.028465185	0.752154887
0.562364280	0.437872052	0.553385794
0.316944152	0.710836232	0.604283392
0.253241092	0.572916031	0.691931725
0.354929805	0.780876040	0.061846159
0.344479710	0.528488874	0.053957056
0.730152845	0.788528502	0.064187035
0.720103800	0.536090791	0.054143645
0.972447574	0.784404814	0.056255147
0.968385279	0.532107830	0.053041480
0.544626772	0.849179566	0.129695967
0.684787750	0.189726606	0.550337553
0.688333154	0.434477895	0.543559134
0.278926790	0.200168267	0.552080870
0.269375801	0.453218758	0.551618993
0.040026322	0.202878639	0.558580816
0.028557133	0.455902785	0.551525176
0.479658604	0.170603529	0.618152678
0.206894070	0.317342460	0.305499703
0.460737914	0.320130020	0.308140874
0.217920363	0.699602127	0.306406826
0.470254958	0.699845433	0.308361202
0.215438589	0.940981209	0.304048985
0.470724016	0.941413164	0.303483218
0.163185745	0.514679432	0.375238746
0.802858233	0.669204235	0.808381736
0.560906887	0.680889845	0.819416881
0.797000229	0.291075408	0.807779074
0.544511080	0.284115672	0.809132516
0.803014100	0.044896532	0.808846235

0.548611939	0.046743177	0.810233653
0.872419596	0.481662154	0.872684360
0.677845597	0.806275249	0.950254619
0.673642814	0.551948011	0.940743864
0.293618500	0.799169242	0.947766900
0.299547493	0.547538400	0.939189911
0.050392188	0.792870045	0.945657969
0.057025149	0.537268400	0.943230569
0.484469771	0.865973175	0.887575150
0.352264166	0.199099883	0.442836016
0.359631956	0.455864549	0.446366429
0.727832615	0.185419708	0.436232716
0.735946715	0.435345680	0.429335952
0.973724186	0.190213472	0.444605261
0.971534669	0.437846065	0.439192295
0.538174152	0.126138791	0.374901116
0.789224386	0.338559747	0.692820847
0.535294592	0.355293095	0.695059657
0.769837737	0.711360455	0.690995574
0.535877407	0.751349926	0.706752241
0.798022270	0.961147964	0.699524820
0.550586760	0.993760049	0.696285546
0.850013614	0.526662588	0.622947276
0.219045117	0.652985394	0.191995218
0.472274035	0.656239748	0.194158450
0.216145933	0.279671520	0.190416336
0.468013197	0.280130476	0.192619592
0.214710176	0.036299773	0.196984664
0.469870061	0.035752822	0.195849478
0.150814921	0.460758746	0.120489180
0.159471676	0.850502133	0.128207043
0.537976027	0.463217020	0.122959085
0.855370939	0.132794797	0.627914011
0.155966088	0.127749637	0.373083323
0.539487004	0.514988720	0.375785530
0.870622754	0.860563636	0.874862552
0.483352572	0.484392464	0.867632210
0.407544941	0.572905123	0.646162510
0.578097999	0.640374243	0.608657837
0.536097169	0.510211885	0.562733114
0.314231634	0.769059837	0.628642797
0.209477425	0.512357533	0.682735860
0.262451172	0.574502766	0.728617609
0.373855531	0.729164958	0.580529451

THF@open site

CONTCAR
1.0

12.6569995880	0.0000000000	0.0000000000
0.0000000000	12.6569995880	0.0000000000
0.0000000000	0.0000000000	26.3959999084

O	Si	Sn	C	H
131	63	1	8	18

Direct

0.339767098	0.652922034	0.066601306
0.343384475	0.815849721	0.002605166
0.265620619	0.840826631	0.093511492
0.470835149	0.815171421	0.080044448
0.352095664	0.511937439	0.992468536
0.237128437	0.471873015	0.074671388
0.447263241	0.472310066	0.078927346
0.708433151	0.661271811	0.064433567
0.853910446	0.814834952	0.072467014
0.665886819	0.843985975	0.108912289
0.844037414	0.502329767	0.057145588
0.654325545	0.470064342	0.095835187
0.989273131	0.657508016	0.059128650
0.054670811	0.841186404	0.093728751
0.032116052	0.470036268	0.096484073
0.731298447	0.310619533	0.551586866
0.691524327	0.147347957	0.491661847
0.766220331	0.117141739	0.584219754
0.566668749	0.175695539	0.570646226
0.720420063	0.479872197	0.489147514
0.757613480	0.507786095	0.584985018
0.505805850	0.613176882	0.590111792
0.267075717	0.319774121	0.542545140
0.163715690	0.149796024	0.561240911

0.361459792	0.160178348	0.595089555
0.153034389	0.494810134	0.548187375
0.323557854	0.464826882	0.606029987
0.036740951	0.324894398	0.565218806
0.974481106	0.143332899	0.599726498
0.970016897	0.518265605	0.595336616
0.334994256	0.308389544	0.316673607
0.188792929	0.331618190	0.245091423
0.149210125	0.210203171	0.324776947
0.158025801	0.420038551	0.333280206
0.489388198	0.327330947	0.247894302
0.521647632	0.217509359	0.331006050
0.503695488	0.427894950	0.334218830
0.345399976	0.696534574	0.316865653
0.175775900	0.817734301	0.306951374
0.160879716	0.629087925	0.349177659
0.514665306	0.819167972	0.307512373
0.530102551	0.632607043	0.351647288
0.345217049	0.942068279	0.304527521
0.171537265	0.005709467	0.350603461
0.520592630	0.010147668	0.347708553
0.685357988	0.660738170	0.831377327
0.800317705	0.665583968	0.745994270
0.857717812	0.778397083	0.826761603
0.872022867	0.567324400	0.825610518
0.558472395	0.744422138	0.764532924
0.501681447	0.753678560	0.858610451
0.501435220	0.566107869	0.819612563
0.671676695	0.305135012	0.812987447
0.832510591	0.167883560	0.813906074
0.854413748	0.363512129	0.849508464
0.516416907	0.165827602	0.823670983
0.483478725	0.364099532	0.844514489
0.674955964	0.027688583	0.812181234
0.858219445	0.980479419	0.855005205
0.491162270	0.964844942	0.846616685
0.656303406	0.678366005	0.942886055
0.693471491	0.834288299	0.008500673
0.781241894	0.834918082	0.917315483
0.575022578	0.873882830	0.929900885
0.673588514	0.506198585	0.997732937
0.783622265	0.517111301	0.913336039
0.574070871	0.498987466	0.909568310
0.300387800	0.674041748	0.931935668
0.171787918	0.836044729	0.944004178
0.364759177	0.869112253	0.908325851
0.179301023	0.502483606	0.935145020
0.366948992	0.498123407	0.892017484
0.047092281	0.665183365	0.938448012
0.987314641	0.847189665	0.898620665
0.988458753	0.482309937	0.897978663
0.364465952	0.324537218	0.441270351
0.330952048	0.148433134	0.497093678
0.257784218	0.160063416	0.405084372
0.463727057	0.144188195	0.421045393
0.340154827	0.493736088	0.503348112
0.267849833	0.498602659	0.409371763
0.472066134	0.502872586	0.427004665
0.701621652	0.313585162	0.429684520
0.854377985	0.169443861	0.427536011
0.663515925	0.125259027	0.392617792
0.858004451	0.453951329	0.414649159
0.665217638	0.501216352	0.390644342
0.010794273	0.315226912	0.432613730
0.050352614	0.121505603	0.405210435
0.058081526	0.511904180	0.410866171
0.669269919	0.350535810	0.686433733
0.833305657	0.329783857	0.750383377
0.833659112	0.228886068	0.663113534
0.855628967	0.438881755	0.668336749
0.510441244	0.306734502	0.748322546
0.488515645	0.274374336	0.651479065
0.494918674	0.472399235	0.686703503
0.648207784	0.698363364	0.676846564
0.805937946	0.836303055	0.688919425
0.843122423	0.645301044	0.648405135
0.530690432	0.870726347	0.684150338
0.432504863	0.691955149	0.689757466
0.682574451	0.011682692	0.694387496

0.873264432	0.020854842	0.657719433
0.501470685	0.064020835	0.650713444
0.345688909	0.658613086	0.182133108
0.194506004	0.641219497	0.251457542
0.162417814	0.759450138	0.171160772
0.170553699	0.550398171	0.162587821
0.496727377	0.641250610	0.252946407
0.524435461	0.765502393	0.174471125
0.524528682	0.556013882	0.163379863
0.342323601	0.287505120	0.179078877
0.177989900	0.157811508	0.188942477
0.155871883	0.344406575	0.146018818
0.506171107	0.157099411	0.188051492
0.532061696	0.347921133	0.150131226
0.341843098	0.025715813	0.191354379
0.157157287	0.967174292	0.152774170
0.519694686	0.967074752	0.147490218
0.994694412	0.825022638	0.998218298
0.012702276	0.496479720	0.997079849
0.996345341	0.164323494	0.501061261
0.969798326	0.468655318	0.499171555
0.176020205	0.992629051	0.251119465
0.511491776	0.988433063	0.247736171
0.844731092	0.991783559	0.756012201
0.508719206	0.026027814	0.750127912
0.565806508	0.434014410	0.555362463
0.293357372	0.689224362	0.604885101
0.267215341	0.546151757	0.700473666
0.354670227	0.780029178	0.060942125
0.344480962	0.527120233	0.053578973
0.730275273	0.787835419	0.063191459
0.720202982	0.535499692	0.053557251
0.972931087	0.784352422	0.055747762
0.968162119	0.531869888	0.052416425
0.544214964	0.847914398	0.128179535
0.686905265	0.189152718	0.549966037
0.691176116	0.433539778	0.545272946
0.282470465	0.194472849	0.549701035
0.273499995	0.446132630	0.551122546
0.044239521	0.197731286	0.555919230
0.032813843	0.450542748	0.552131772
0.481456876	0.169898927	0.616902351
0.208360896	0.317634344	0.305551440
0.461950511	0.320336580	0.307942808
0.219521448	0.696981251	0.305721790
0.471855432	0.697938621	0.307105601
0.217491627	0.938881934	0.303296924
0.472897798	0.940100610	0.301829100
0.162404880	0.513534546	0.375551999
0.803883553	0.668970823	0.807207108
0.561402559	0.680610836	0.817501485
0.798008502	0.290863454	0.806452513
0.545787573	0.285630971	0.806265354
0.802004755	0.043631699	0.808765411
0.548306167	0.045928255	0.807979584
0.872402728	0.481831640	0.871915638
0.676128328	0.804691374	0.949363530
0.672432125	0.551033795	0.939935505
0.294282407	0.798126996	0.946764469
0.299731791	0.546710312	0.938671887
0.050825816	0.792816997	0.945076168
0.056723367	0.537415624	0.942744493
0.484328896	0.867189765	0.885961294
0.354316950	0.196395755	0.441066235
0.360682994	0.453033179	0.445797473
0.728772283	0.188885450	0.436020404
0.735695541	0.437464952	0.431748927
0.976647377	0.192724094	0.441829681
0.973770261	0.437564731	0.439447850
0.541736841	0.125054821	0.372879475
0.795875251	0.336245000	0.691398084
0.540462136	0.354976743	0.692320347
0.774537742	0.711641729	0.689851582
0.541054130	0.749173522	0.703393877
0.800944388	0.962593198	0.699721515
0.555393934	0.993999243	0.694674492
0.855356932	0.527167201	0.623575330
0.218735844	0.652101099	0.191415235
0.472173631	0.654684961	0.192992970

0.216446251	0.279910356	0.190258235
0.467439294	0.279276341	0.191793069
0.214585066	0.036213081	0.196361512
0.469526440	0.034726117	0.193868309
0.150135145	0.460189253	0.120007388
0.160094991	0.850686312	0.127800718
0.538648307	0.461769640	0.122165583
0.860038340	0.130079076	0.625873864
0.157199442	0.125376329	0.371377856
0.541909635	0.515145242	0.375610113
0.869945288	0.859473348	0.874616921
0.482923418	0.482889295	0.866839826
0.403134406	0.569809616	0.645231664
0.337417632	0.787040591	0.583040833
0.242816225	0.845786750	0.559766591
0.147309989	0.772928774	0.569625318
0.182149485	0.709474862	0.615461290
0.239297301	0.436637700	0.714100897
0.165636763	0.445122808	0.760143340
0.161367670	0.564114213	0.772933245
0.257890433	0.609653413	0.746777773
0.571123004	0.637382209	0.606243134
0.536233008	0.506020606	0.565025508
0.254416913	0.860335469	0.519149065
0.134134158	0.719081819	0.537575424
0.175692394	0.755543590	0.650880814
0.397392213	0.761428773	0.555796742
0.375430167	0.831861496	0.613732874
0.143203914	0.632835746	0.619821370
0.074103214	0.817088187	0.576673388
0.231814370	0.922780156	0.578250170
0.197872460	0.399541855	0.791985571
0.163938552	0.578482091	0.813951790
0.250543803	0.692099452	0.734694242
0.312823206	0.394373298	0.723098993
0.203387395	0.402126431	0.680187941
0.330566913	0.600024700	0.769147933
0.088718057	0.600086272	0.758042932
0.086528309	0.414264023	0.751712799

o-Glu@open site

CONTCAR
1.0

12.6569995880	0.0000000000	0.0000000000
0.0000000000	12.6569995880	0.0000000000
0.0000000000	0.0000000000	26.3959999084

O	Si	Sn	C	H
135	63	1	6	14

Direct

0.339135677	0.641973495	0.072908223
0.345186740	0.794401407	0.003388950
0.256662667	0.829941094	0.091491744
0.464264333	0.808902681	0.083935730
0.351591170	0.505432665	0.996100187
0.240039304	0.459238112	0.078666143
0.450598836	0.464004308	0.080676988
0.712127388	0.654263020	0.066978954
0.851103187	0.813274264	0.069670446
0.663382351	0.840125084	0.107111976
0.845616341	0.492886037	0.061549645
0.656184554	0.468195856	0.102315649
0.985116720	0.651887417	0.061443739
0.046740592	0.836004615	0.097849183
0.034932986	0.468513459	0.101672202
0.730182648	0.295683980	0.556195378
0.700198412	0.128606066	0.497728109
0.778282285	0.108250171	0.591167569
0.574643373	0.154098749	0.575400829
0.704487741	0.464275390	0.494774491
0.755801320	0.485888660	0.590999007
0.574554861	0.608036816	0.569226444
0.283717513	0.308140457	0.573969901
0.175707340	0.135470003	0.556555986
0.370313525	0.123404808	0.597020566
0.159992084	0.473911256	0.558476090
0.342704713	0.496965975	0.607325852

0.040580884	0.300115705	0.561373115
0.989577413	0.111885548	0.598722219
0.967583716	0.483493924	0.599419832
0.346391797	0.283104390	0.316585392
0.191689000	0.323610485	0.250789583
0.156664521	0.206193790	0.332163006
0.186882272	0.414586216	0.339616954
0.500071704	0.320208997	0.249758199
0.539783239	0.212256253	0.333098769
0.500160933	0.420107275	0.336703002
0.345028460	0.687438428	0.321660519
0.175584212	0.810645998	0.310336232
0.157970548	0.622093320	0.352167875
0.514164686	0.812100649	0.311951071
0.530384302	0.623375297	0.354904264
0.344928175	0.932819784	0.303568393
0.174898520	0.998685122	0.351315051
0.515000880	0.004675061	0.349886656
0.683953106	0.648162305	0.832753837
0.803174555	0.639459431	0.748629630
0.860684574	0.758166254	0.827630758
0.867426217	0.547142565	0.830610573
0.551997244	0.705421090	0.761539221
0.498094589	0.741315067	0.854194462
0.503184021	0.544526100	0.825677693
0.674778938	0.285314143	0.816365778
0.834245563	0.147451714	0.821822226
0.854175925	0.344442844	0.855944037
0.514478743	0.150908157	0.815937519
0.485350251	0.342571437	0.847549438
0.674264550	0.011695190	0.813841999
0.852822959	0.957578063	0.858661711
0.487658858	0.954015613	0.846663594
0.647518814	0.658746183	0.944154024
0.686474562	0.819567859	0.007189484
0.787131846	0.804093421	0.919963121
0.581647158	0.857349098	0.924861491
0.672651112	0.493911654	0.003427332
0.781225026	0.498861820	0.918800712
0.573084772	0.472708136	0.915338993
0.303195089	0.657820761	0.930181324
0.175529465	0.820796609	0.944212317
0.369354129	0.853821993	0.910058379
0.179511100	0.487935066	0.939069688
0.367331028	0.473630995	0.896964192
0.048183370	0.651420474	0.942480922
0.990088940	0.830213964	0.899654329
0.986236989	0.467356682	0.903608382
0.343113303	0.321526885	0.430548996
0.349110097	0.173935324	0.502204537
0.247569025	0.138117015	0.417170137
0.457610160	0.145133942	0.419669092
0.347465575	0.453498989	0.509192646
0.250719547	0.509140193	0.423947632
0.461375147	0.494633049	0.428921759
0.694844127	0.296482533	0.435806394
0.851574838	0.157506779	0.429627508
0.659215868	0.106933214	0.399579823
0.851034105	0.438819736	0.424692929
0.658167362	0.485250890	0.396530032
0.007920117	0.300088227	0.438074261
0.042385641	0.108947702	0.402613193
0.045965657	0.492881238	0.405740321
0.686113000	0.276689470	0.689277709
0.842562377	0.304298133	0.756546557
0.876203120	0.209807128	0.667719126
0.845818460	0.416488796	0.674850523
0.521590114	0.313908726	0.749433696
0.490590632	0.236504242	0.658894062
0.560066104	0.436450362	0.667581141
0.664334714	0.690074503	0.677228034
0.834458113	0.811768234	0.695047736
0.854436636	0.620995343	0.651843727
0.516805708	0.836841702	0.686069429
0.459002763	0.635371208	0.679133415
0.686558425	0.962172806	0.694843471
0.879964352	0.999099016	0.661055267
0.513216734	0.026630482	0.649338245
0.347091436	0.667125881	0.186791867

0.195242539	0.636835217	0.254358411
0.160726964	0.757146657	0.173846066
0.178969920	0.547896028	0.165156543
0.497152388	0.637109876	0.256205887
0.533646345	0.755808353	0.175774097
0.515233517	0.545761824	0.167832881
0.344779700	0.283018410	0.184320688
0.180455387	0.152342916	0.192423761
0.156409249	0.341505319	0.152478278
0.506767809	0.149201483	0.190513477
0.533577442	0.339325637	0.151605815
0.342445970	0.018073555	0.194499642
0.159765288	0.963496149	0.152245492
0.519831717	0.958966434	0.150219440
0.001746294	0.817061365	0.999989569
0.015671883	0.485670626	0.002125621
0.000549579	0.137281537	0.500412047
0.985977113	0.461571813	0.501475692
0.173705280	0.982471526	0.251466513
0.513941169	0.980158687	0.250040859
0.846015394	0.978921711	0.759042561
0.514131129	0.998866975	0.749059379
0.556751609	0.408142745	0.561355472
0.899035394	0.709283948	0.492475897
0.096261710	0.790624022	0.462298781
0.175564572	0.702949643	0.588375688
0.309608340	0.710207582	0.488814056
0.383717716	0.733494222	0.591789484
0.550255179	0.812823057	0.539182544
0.351327926	0.768286884	0.063009433
0.345365971	0.517818630	0.057093311
0.728294909	0.781225622	0.062278889
0.722143590	0.527744651	0.057998605
0.971103311	0.778980851	0.056894816
0.969309986	0.524995029	0.056283310
0.544227183	0.840774953	0.129610047
0.694650054	0.171232760	0.555843890
0.687588274	0.417093545	0.551411092
0.294490725	0.185450971	0.557537377
0.284342498	0.433940411	0.562702179
0.052317027	0.172402650	0.554198980
0.038588770	0.427674502	0.555934250
0.488899022	0.136491030	0.620793283
0.220504940	0.307661563	0.310172915
0.471792459	0.309388787	0.309401929
0.219369233	0.690102339	0.309227943
0.471531004	0.690763414	0.310832411
0.217470363	0.931156456	0.303719103
0.472426683	0.932420850	0.303664863
0.160870016	0.507828176	0.379688770
0.803567290	0.649095535	0.809543908
0.559192657	0.658960938	0.817891240
0.801346779	0.270162523	0.812599778
0.549012542	0.272816032	0.806236684
0.801546931	0.024930321	0.812717140
0.548338413	0.029111363	0.806527972
0.870314121	0.463829517	0.877476156
0.675313592	0.784161687	0.948368251
0.668897390	0.531661212	0.944434583
0.297310024	0.781143785	0.946756244
0.300189227	0.531558037	0.940900087
0.054187033	0.779247642	0.946728706
0.057521392	0.523773730	0.946721613
0.485272020	0.852384925	0.883789778
0.349551141	0.195084065	0.441905409
0.349865496	0.444982916	0.447895736
0.726894736	0.172013551	0.441246510
0.727077365	0.420781314	0.437851578
0.974608839	0.176018208	0.443106622
0.972146928	0.422919840	0.442668289
0.542131007	0.117856435	0.375034183
0.811315298	0.302131921	0.696947098
0.563295722	0.319547206	0.690982997
0.788503408	0.691838145	0.692884386
0.546301246	0.715634048	0.700429916
0.810829580	0.936857045	0.703229487
0.558566570	0.955638707	0.695228755
0.855573833	0.501231968	0.629325449
0.220533118	0.651876211	0.194441050

0.473007262	0.650812328	0.196110219
0.218737766	0.274557859	0.195149496
0.470984846	0.272216380	0.194189116
0.214899242	0.029505303	0.197542652
0.470187843	0.026945559	0.196422845
0.153271556	0.455367535	0.124283627
0.156223908	0.845219851	0.128675669
0.538190305	0.455275625	0.125472948
0.878792286	0.108847506	0.629780173
0.156692609	0.114229776	0.375340223
0.536923349	0.505710840	0.378955334
0.871637404	0.836637795	0.876435101
0.483037919	0.460090965	0.871519506
0.490240276	0.540354252	0.622045994
0.973869801	0.743454933	0.529780447
0.085839346	0.733954012	0.508973420
0.168687776	0.774996221	0.546277225
0.275605857	0.796114028	0.519917786
0.363453090	0.818372250	0.558779836
0.463336349	0.852418840	0.532503724
0.558840156	0.684669197	0.565228760
0.517693162	0.438030005	0.532571197
0.966223240	0.699276567	0.565563202
0.956249714	0.827191830	0.537451148
0.103260152	0.649134338	0.502027869
0.142348394	0.850727320	0.562788129
0.264872491	0.868423462	0.496896267
0.338501275	0.890115917	0.580303907
0.171159238	0.630121410	0.575715780
0.260677516	0.706920385	0.460208863
0.453694075	0.921133637	0.506361127
0.890933037	0.632970512	0.495542943
0.031285688	0.776809752	0.443286359
0.313308328	0.710195303	0.603787124

Glucopyranose@open site

```
CONTCAR
   1.00000000000000
    12.6569995879999997    0.0000000000000000    0.0000000000000000
     0.0000000000000000   12.6569995879999997    0.0000000000000000
     0.0000000000000000    0.0000000000000000   26.3959999084000003
   O    Si   Sn   C    H
 135  63   1   6  14
Direct
  0.342121866  0.654089878  0.067953517
  0.342044866  0.817580150  0.004209586
  0.268444436  0.842128742  0.095910185
  0.472456396  0.817453444  0.081234300
  0.352870405  0.512377625  0.993697366
  0.240646004  0.472109342  0.076810793
  0.450771831  0.474233689  0.079521866
  0.713026277  0.660988594  0.066720584
  0.857186093  0.815516923  0.075073109
  0.668072761  0.844924073  0.110151165
  0.846902736  0.500597983  0.058656818
  0.657318941  0.470628268  0.098444970
  0.991089068  0.657357674  0.060450075
  0.057643798  0.842540424  0.093628035
  0.034830674  0.470065778  0.097456384
  0.723550803  0.309179039  0.554851224
  0.689786375  0.145905639  0.494350042
  0.768177318  0.118177608  0.586730433
  0.565613145  0.168322456  0.574132391
  0.720526881  0.476839342  0.490236939
  0.751595732  0.507203102  0.586368179
  0.529629914  0.632426138  0.584015118
  0.277572640  0.325663326  0.553340717
  0.165100009  0.155026957  0.561987842
  0.360500094  0.153567424  0.598400571
  0.151746535  0.494320605  0.550310951
  0.319439345  0.488659862  0.610027040
  0.033492230  0.324458805  0.564892011
  0.977146297  0.140829909  0.601555374
  0.965184124  0.517423007  0.594122443
  0.337449791  0.300966363  0.316497269
  0.187560669  0.328414257  0.246493111
  0.148065648  0.214024278  0.328795281
```

0.167854202	0.423651027	0.333325583
0.493350281	0.327529146	0.248955504
0.527269146	0.219026751	0.332648497
0.499580564	0.428554287	0.335674538
0.345661806	0.699833948	0.318307249
0.174500402	0.819972911	0.308365485
0.160965559	0.631814153	0.350493471
0.515652111	0.821127942	0.310109971
0.529436180	0.633363933	0.353297073
0.344373650	0.942908525	0.305397828
0.170789785	0.007973637	0.351869444
0.517101825	0.011610789	0.350346168
0.685254306	0.663617989	0.832375048
0.800355637	0.660576947	0.747583090
0.858390318	0.778672141	0.827229689
0.870573500	0.566778551	0.828507762
0.554955003	0.743963348	0.766060273
0.501104363	0.754165823	0.860277090
0.503304101	0.564949458	0.821045310
0.673238519	0.295694661	0.815106055
0.841275259	0.168238129	0.813621692
0.854114654	0.362141011	0.851717566
0.511134856	0.164705518	0.821544400
0.487443773	0.362541173	0.847372858
0.678001992	0.035237776	0.813665767
0.861129343	0.981089924	0.855051655
0.497103509	0.966414542	0.850791069
0.662368912	0.677249435	0.944868581
0.699304725	0.833711181	0.009981116
0.781292703	0.837960258	0.917450445
0.575180853	0.870434204	0.933099879
0.674197281	0.505449896	0.000375995
0.785461662	0.512873685	0.916490195
0.575532423	0.499812419	0.911361848
0.302241990	0.674900822	0.933598209
0.171634720	0.836397051	0.944825989
0.365348673	0.870993593	0.910226022
0.180991012	0.502798055	0.936093497
0.368918301	0.497762764	0.893537797
0.047279440	0.664932899	0.938775504
0.987805659	0.847808347	0.899301161
0.990063353	0.480069405	0.899209807
0.358128641	0.328449500	0.443313733
0.334070512	0.156574362	0.500697634
0.255138858	0.158888412	0.409424432
0.462456946	0.150188805	0.422470819
0.340845024	0.496470938	0.506743508
0.261574975	0.504085111	0.414124995
0.468088606	0.505223319	0.428440828
0.702252173	0.310826713	0.431121374
0.852659636	0.163903097	0.430158471
0.661990063	0.121649364	0.395622337
0.855332152	0.453941216	0.414800897
0.661422762	0.498919763	0.392313674
0.005906760	0.312903280	0.433639853
0.048563133	0.118527211	0.407283786
0.053056879	0.508811585	0.407608961
0.671694008	0.336924546	0.686025003
0.832286211	0.333573369	0.752474839
0.841073779	0.221783269	0.667992550
0.854331441	0.432598822	0.667078896
0.512809112	0.316004012	0.750381519
0.486239393	0.267855726	0.654600200
0.508950067	0.469685324	0.680409442
0.649313685	0.697620551	0.678825447
0.812120067	0.830845402	0.690542487
0.840825970	0.640562342	0.649297997
0.522492772	0.863155173	0.683250964
0.435438502	0.677176834	0.691525536
0.681515539	-0.001838619	0.694130344
0.873483797	0.014152522	0.657247642
0.500124181	0.057696526	0.654234433
0.346958974	0.660846163	0.183875159
0.195194289	0.643623943	0.252877822
0.161757013	0.758861901	0.171832767
0.172799571	0.549559285	0.164654768
0.497978143	0.644037947	0.254614371
0.527361786	0.765266256	0.175323205
0.524578464	0.555256815	0.165492215

0.343337630	0.287824971	0.181386531
0.178899569	0.157219625	0.188897786
0.156892790	0.344079552	0.147191889
0.506888506	0.157200838	0.189282429
0.532609700	0.347815436	0.150753274
0.342536877	0.024474734	0.193224862
0.156493900	0.966909916	0.154331879
0.522216871	0.967430828	0.149773717
0.994360718	0.824028722	0.998819457
0.015006034	0.496663398	0.997907146
0.996153503	0.161211854	0.502740669
0.972392788	0.470165814	0.498259591
0.174364323	0.995073115	0.252554398
0.512340476	0.990168174	0.250105063
0.844527057	0.991841494	0.756037223
0.511624224	0.013402290	0.753124274
0.561006423	0.436813288	0.553319891
0.119156941	0.728265242	0.707123509
0.310635897	0.711401735	0.601491215
0.052058409	0.834022460	0.622705726
0.089620502	0.882839519	0.519261230
0.311066720	0.885588814	0.488090259
0.391364653	0.878469848	0.587238789
0.356111804	0.781449227	0.062338360
0.346750524	0.528241057	0.054671468
0.733897392	0.787843940	0.065111391
0.723355951	0.534873309	0.055704163
0.974995078	0.784361703	0.056702427
0.970734514	0.531431508	0.053243815
0.546472959	0.848979428	0.129422080
0.684745253	0.185649433	0.552908369
0.688659421	0.431886508	0.546100770
0.284867996	0.198801668	0.554124671
0.273652855	0.453042519	0.555497288
0.043844594	0.196964684	0.557266181
0.031118964	0.450214746	0.552224464
0.479994443	0.162676681	0.620393344
0.210786641	0.316979794	0.306666991
0.464247330	0.319140810	0.308714825
0.219835158	0.699805500	0.307039338
0.472281839	0.700193657	0.308794753
0.216571371	0.940849905	0.304551503
0.472281843	0.941351385	0.303899491
0.161831803	0.515629576	0.376009081
0.804644823	0.668405430	0.808807520
0.560523877	0.680403658	0.819463544
0.800217057	0.289621652	0.808236171
0.546416829	0.284889438	0.807815255
0.805768054	0.045444142	0.809156438
0.550344010	0.045008280	0.810070288
0.873316541	0.480129692	0.874146242
0.679461958	0.804156095	0.950967927
0.674416668	0.549305181	0.942339402
0.294122144	0.799230921	0.947996701
0.301107827	0.547212821	0.939624327
0.050849470	0.792606295	0.945501837
0.058379566	0.537203578	0.943181150
0.485294596	0.866933018	0.888688951
0.352894562	0.199894555	0.443403755
0.356738154	0.456779659	0.448226083
0.727647055	0.185859066	0.438490758
0.734052332	0.435360120	0.432362861
0.974547205	0.189823188	0.443760077
0.971568589	0.436649176	0.438871591
0.541142856	0.126121587	0.374926576
0.798589000	0.330943418	0.693189625
0.543925448	0.350303661	0.692687189
0.776602586	0.707746715	0.691713126
0.540143206	0.743684698	0.705229532
0.802836915	0.957114081	0.700288983
0.554149967	0.984828645	0.696902106
0.852667013	0.523600118	0.623883077
0.219844602	0.652937618	0.192740875
0.473681241	0.655724870	0.194445601
0.216995876	0.279166748	0.191286380
0.469074146	0.279667414	0.192832649
0.214990992	0.035991716	0.197510861
0.470606766	0.034930296	0.195775804
0.152150334	0.460212902	0.121431858

0.161038955	0.850932430	0.128771839
0.540361691	0.462508643	0.123423439
0.863196829	0.126721472	0.628173822
0.156136198	0.126406673	0.374094934
0.538679029	0.515618662	0.376931102
0.870990771	0.860235808	0.874922308
0.484486255	0.482411958	0.868370402
0.433790264	0.572803124	0.637019508
0.355629753	0.785363197	0.564513464
0.223359954	0.756982749	0.630877174
0.135572364	0.787760314	0.593566743
0.175546687	0.863762861	0.553263978
0.270710956	0.816037856	0.526004208
0.183634731	0.677422352	0.670359298
0.599972969	0.639511811	0.599269888
0.537751661	0.511748936	0.551924277
0.419891362	0.740277713	0.546883222
0.107136851	0.715004407	0.574627606
0.199511241	0.938290931	0.571921306
0.245908354	0.744112595	0.505734084
0.252559875	0.827537340	0.650925018
0.250965853	0.644629750	0.691578517
0.066930339	0.769755430	0.688145143
0.000391655	0.863686680	0.599252892
0.118273852	0.919819078	0.489834511
0.346148145	0.944235788	0.505437717
0.450715700	0.864088876	0.610039406
0.141608980	0.611815117	0.651414494

Ethanol@closed site

CONTCAR
1.0

12.6569995880	0.0000000000	0.0000000000
0.0000000000	12.6569995880	0.0000000000
0.0000000000	0.0000000000	26.3959999084

O	Si	Sn	C	H
130	63	1	4	12

Direct

0.327592492	0.654770434	0.068384409
0.348009020	0.812190056	0.001667105
0.235604286	0.839491427	0.084150076
0.445916146	0.823083699	0.088307500
0.346047401	0.507957399	0.996256173
0.229679421	0.471600354	0.077708520
0.440186858	0.478719294	0.082831621
0.707094193	0.668040931	0.069257453
0.836936057	0.833331645	0.062877528
0.650239289	0.854317546	0.104860313
0.837158859	0.503379285	0.062685519
0.647259712	0.480189472	0.101432301
0.967649221	0.668353081	0.060236439
0.028148975	0.850320041	0.100124680
0.026686750	0.489769518	0.103331409
0.707863331	0.323618650	0.559922159
0.676045835	0.159059852	0.499202251
0.750225604	0.131231070	0.592823267
0.550130427	0.182769537	0.576654732
0.682195902	0.486591667	0.497005314
0.755637348	0.518712401	0.588204563
0.552011132	0.460034341	0.575746179
0.275007963	0.314215004	0.557584584
0.158508360	0.144506633	0.564011514
0.352944195	0.139354363	0.601090431
0.146935716	0.482870102	0.554515362
0.320593327	0.489986002	0.606648028
0.027391108	0.313480288	0.559975863
0.960926235	0.129738525	0.591457069
0.963569820	0.492234290	0.603807986
0.329089224	0.323312521	0.323468268
0.192640096	0.338160336	0.246495768
0.150388569	0.214915022	0.324238807
0.144978181	0.424509168	0.333695441
0.472997993	0.332099855	0.249839365
0.513303339	0.224607572	0.333777428
0.503665805	0.435378015	0.333958954
0.335195124	0.700017750	0.320485502
0.168436408	0.824086308	0.311175793

0.148983151	0.632683218	0.350598544
0.502615631	0.825770080	0.311396807
0.517822325	0.639517486	0.355932683
0.335809320	0.953447223	0.309281677
0.155365646	0.012304260	0.350559115
0.518372476	0.016157048	0.348763406
0.672745705	0.658411622	0.818673670
0.826009691	0.663836658	0.748866916
0.844859302	0.771836698	0.833971143
0.856915295	0.561484277	0.832615793
0.521559417	0.678622067	0.749344289
0.488948584	0.747522712	0.842527568
0.497086138	0.544590652	0.825225472
0.672195196	0.305937767	0.820047498
0.825518906	0.161267772	0.820223510
0.861057043	0.355661660	0.852488101
0.522737324	0.157772899	0.827800393
0.481987983	0.347832233	0.855682492
0.673882365	0.012682221	0.816367567
0.859471560	0.974706948	0.858286381
0.480576962	0.956784964	0.841064453
0.664836764	0.669901788	0.936514497
0.662431300	0.824236870	0.005957729
0.785627306	0.837385714	0.925809562
0.575812697	0.856660724	0.917283416
0.666588306	0.513621390	0.003402003
0.770823061	0.491448879	0.917796612
0.560586989	0.492867559	0.918689430
0.296837628	0.665868878	0.934034050
0.174242154	0.832846761	0.944435418
0.365128011	0.854119539	0.904952109
0.171343386	0.496946335	0.940565526
0.356931776	0.484685540	0.896439373
0.045789089	0.664341688	0.945019007
0.987975121	0.840794861	0.900445521
0.979237974	0.483464181	0.904579520
0.344391763	0.321657300	0.441157579
0.325467080	0.151020572	0.501408756
0.227572873	0.158725128	0.413752049
0.438001394	0.140784144	0.418783128
0.329061598	0.483301878	0.505010068
0.245080695	0.499303669	0.413658589
0.450843394	0.501488447	0.426401943
0.691598237	0.322065115	0.435595989
0.837292790	0.169290766	0.435533911
0.645201087	0.134485900	0.400526017
0.837775946	0.472815782	0.427231967
0.649044514	0.510415733	0.397851914
0.984338164	0.322269827	0.437004983
0.022421855	0.137413338	0.395368189
0.037027035	0.514667451	0.409634560
0.668074250	0.302118123	0.690519094
0.831153333	0.318843067	0.754338145
0.858561814	0.241385788	0.661777377
0.824114740	0.444920391	0.676417887
0.509675384	0.310087234	0.757214367
0.472897887	0.248940066	0.664459825
0.518993974	0.452889770	0.684241354
0.674629629	0.720133960	0.684141159
0.846226215	0.836010039	0.692302942
0.858880997	0.644033611	0.651149452
0.511915743	0.855501890	0.696213245
0.485498041	0.681167364	0.648146570
0.682823598	0.972679496	0.694277585
0.868212581	0.030712133	0.661466956
0.507278562	0.041570157	0.649111688
0.341467887	0.681295276	0.186076194
0.186160207	0.653211892	0.252891243
0.153426841	0.764705300	0.169621259
0.177747369	0.554983377	0.166377842
0.489765197	0.649932742	0.256508827
0.528211355	0.772249639	0.177350298
0.512422264	0.563167274	0.167645752
0.334906995	0.287950367	0.176674694
0.168010950	0.162373737	0.191548765
0.144344613	0.350871861	0.149650946
0.501171529	0.163666427	0.191185653
0.526206195	0.355849236	0.153744400
0.334254503	0.034549803	0.192357227

0.153745577	0.972304583	0.151386514
0.513630152	0.974787295	0.150372177
0.001654317	0.833542466	0.000784241
0.006914333	0.499269515	0.003593492
0.007531846	0.153650373	0.494942605
0.967700541	0.478383631	0.504016101
0.169527948	0.991995454	0.250782698
0.500442207	0.992903948	0.249996960
0.843344748	0.994080842	0.758787870
0.517988443	0.033294022	0.748764157
0.453716069	0.637695193	0.550605476
0.329731226	0.575393736	0.700799644
0.339420646	0.781950951	0.061088789
0.336054146	0.528551638	0.056646440
0.714911878	0.794740021	0.060386956
0.715060294	0.542023480	0.058744162
0.958422720	0.795849800	0.055812616
0.959199071	0.540699363	0.057207618
0.533715248	0.855578959	0.130295008
0.670530558	0.200265214	0.557571888
0.671563625	0.448210090	0.555982888
0.278840393	0.186621115	0.556412160
0.268554866	0.441281378	0.556531131
0.039514277	0.186829969	0.552428246
0.025785521	0.441211462	0.555948198
0.472141892	0.153265730	0.623631597
0.204806104	0.325583518	0.307202727
0.454962969	0.327948362	0.310432732
0.209795058	0.702917755	0.308496237
0.461503774	0.704395294	0.310643673
0.208216116	0.945281506	0.305037707
0.463535100	0.946986079	0.304651141
0.144513503	0.516727269	0.376588047
0.799350441	0.664051116	0.808658957
0.545934081	0.657913923	0.808354080
0.797231734	0.284845352	0.811389148
0.547034621	0.280194789	0.814167976
0.799983323	0.036832333	0.812955439
0.549333990	0.039641336	0.808406055
0.865354955	0.473184824	0.877040684
0.671709538	0.796690226	0.946118474
0.666119397	0.542346954	0.943502069
0.295526147	0.791248560	0.946438253
0.292345107	0.539160311	0.942310274
0.052785359	0.792149782	0.947900712
0.050624672	0.536471665	0.948501587
0.478899270	0.853015304	0.876740277
0.333774954	0.193581581	0.443478614
0.341264755	0.449025899	0.445767581
0.713262200	0.196729600	0.443085015
0.715398252	0.447823107	0.440697402
0.961609423	0.196153387	0.441378862
0.957194567	0.446688056	0.444530338
0.528530836	0.130697876	0.375445724
0.793851078	0.327081650	0.695608735
0.542615354	0.331584066	0.698304236
0.800590932	0.715995669	0.693975747
0.548770308	0.732082427	0.694994092
0.808736026	0.957052469	0.702193141
0.554988325	0.975791335	0.696601391
0.850587785	0.523653865	0.629414082
0.214719430	0.663405716	0.193196490
0.467601329	0.666218817	0.196425021
0.210108042	0.283637077	0.191336095
0.458844364	0.284769744	0.193062574
0.206634432	0.040384509	0.196606383
0.461840749	0.042123284	0.196259245
0.144994989	0.467723131	0.124193609
0.143586457	0.855928957	0.126317397
0.530660808	0.470431596	0.126161516
0.857380927	0.135406479	0.626942396
0.139553130	0.131663531	0.370992213
0.528977036	0.520770252	0.378119051
0.868443847	0.855025709	0.879481673
0.475222886	0.469311267	0.874171853
0.462160885	0.536052525	0.626390457
0.383581549	0.726668596	0.541077495
0.268801838	0.493861407	0.725993574
0.402237952	0.771587610	0.488576114

0.213721260 0.535971999 0.772921383
0.280497849 0.628201365 0.687886417
0.438208818 0.583394229 0.524993002
0.300880373 0.700742245 0.545730293
0.402040929 0.783977985 0.570983768
0.327035666 0.433053762 0.736022532
0.211982995 0.459099859 0.699015617
0.348219663 0.838216126 0.481736660
0.387384444 0.711916029 0.459071636
0.483828932 0.799969852 0.484415084
0.172689170 0.471349239 0.792734325
0.154199675 0.596182287 0.763254344
0.271234423 0.570917845 0.799214125

H2O@closed site

CONTCAR
1.0
12.6569995880 0.0000000000 0.0000000000
0.0000000000 12.6569995880 0.0000000000
0.0000000000 0.0000000000 26.3959999084
O Si Sn H
130 63 1 4
Direct
0.327071309 0.653961539 0.067985825
0.345443428 0.813130498 0.001634917
0.236551568 0.839003742 0.085478015
0.446883321 0.821184635 0.087209888
0.346117437 0.505434513 0.996763289
0.228725135 0.471118331 0.077957101
0.438739628 0.477305561 0.083951861
0.713265717 0.663732409 0.076660261
0.837538660 0.831235170 0.062699623
0.650397480 0.852872312 0.105304651
0.837044418 0.497315079 0.061446123
0.646072924 0.472501397 0.099928759
0.965075374 0.664433002 0.059690200
0.028871231 0.842569709 0.102198005
0.025783774 0.486988217 0.103263639
0.693509400 0.329683989 0.563758135
0.675463915 0.165842295 0.500889242
0.742742121 0.136595041 0.595325768
0.543188870 0.180279046 0.576349378
0.679811895 0.488386035 0.497362167
0.749561965 0.524318457 0.588978052
0.544310749 0.480126739 0.572582841
0.264612913 0.308903396 0.552571535
0.152239397 0.140540764 0.564697981
0.347193390 0.141083822 0.602678180
0.145372346 0.482322961 0.561468065
0.323968738 0.462781161 0.612062395
0.024172589 0.313727349 0.560904860
0.951852262 0.129981101 0.588223875
0.955754042 0.492843539 0.603930533
0.325832248 0.323118031 0.323478788
0.189235955 0.338379860 0.246434122
0.147739276 0.214852795 0.323908210
0.143245369 0.425294578 0.334185183
0.471356422 0.330531865 0.250389367
0.509963572 0.225440413 0.335055649
0.498858482 0.435958147 0.334992558
0.335977763 0.702766836 0.317207158
0.166628167 0.823679626 0.312415987
0.155700475 0.632142365 0.353058070
0.504739165 0.825521469 0.312169015
0.512759507 0.639638186 0.357505858
0.335362673 0.951096117 0.310101628
0.157365233 0.013872904 0.352407098
0.517231405 0.017031139 0.348880887
0.672131836 0.656268477 0.808575153
0.847781718 0.674914777 0.752091110
0.831981421 0.774012029 0.839530289
0.851724625 0.564050496 0.835136354
0.505490065 0.674585998 0.746662557
0.495496184 0.750913620 0.840082049
0.495783210 0.546143770 0.824904501
0.673491359 0.307312638 0.819043040
0.826613009 0.162655711 0.819888592

0.862413406	0.357258141	0.851526797
0.523950577	0.159131527	0.828399956
0.485717118	0.349441111	0.856459618
0.674906492	0.014051694	0.817781329
0.861280560	0.977319300	0.859149575
0.482378840	0.959275842	0.843775630
0.676859200	0.670525968	0.930553079
0.660551250	0.806968868	0.007703394
0.782797575	0.850335598	0.930087388
0.572999835	0.852102101	0.919662714
0.665719986	0.526145875	0.003796726
0.772789955	0.484666526	0.920312464
0.562744975	0.498074263	0.918351829
0.296658516	0.664192855	0.935181081
0.173983902	0.832331479	0.942917347
0.364991903	0.849374115	0.903655887
0.171926007	0.495353669	0.940450847
0.358792841	0.484572977	0.896849990
0.046875220	0.663564742	0.945931256
0.984276056	0.839538991	0.902378559
0.980435729	0.484600455	0.904004872
0.346669525	0.320065320	0.446930021
0.320989311	0.142127171	0.502274811
0.220485151	0.167006835	0.415413916
0.429757863	0.138313428	0.418414801
0.324444503	0.485570133	0.509117067
0.238678396	0.490965575	0.417593062
0.445067823	0.502022922	0.427979946
0.692861736	0.324717671	0.435281277
0.833375156	0.168833837	0.435239762
0.638450921	0.136387601	0.403144121
0.835622787	0.480434895	0.429408878
0.644345939	0.511027515	0.398686290
0.977128565	0.325260401	0.435281515
0.017754797	0.139260605	0.393995136
0.033266634	0.517664313	0.408781052
0.667300820	0.301152885	0.691528380
0.832729816	0.319070250	0.753506541
0.859193623	0.250338882	0.659151077
0.816707253	0.450861901	0.677726746
0.508321226	0.310492396	0.757732630
0.471828997	0.249691501	0.664862096
0.519217253	0.453084141	0.684669077
0.682049811	0.728612959	0.699545383
0.851480365	0.842518270	0.689774036
0.854344308	0.647936046	0.652257860
0.512620568	0.855017841	0.695761740
0.518411875	0.677157223	0.644233406
0.682441831	0.974231899	0.693989515
0.866494179	0.040656753	0.663492322
0.503190279	0.042416945	0.650784671
0.342814595	0.670976996	0.188269302
0.182948470	0.650345623	0.254216075
0.158348724	0.762219071	0.171308443
0.175578311	0.552004516	0.167155892
0.495553643	0.648812532	0.257077813
0.526194274	0.768444061	0.176524788
0.516935527	0.558994949	0.168182582
0.332730532	0.285210192	0.177649930
0.165525869	0.160720319	0.192232803
0.143379420	0.348094404	0.149163887
0.499161512	0.160564452	0.191825613
0.523913920	0.351461381	0.154002652
0.332056105	0.032455921	0.192680314
0.150960386	0.969306290	0.153059587
0.511349559	0.971547604	0.150503054
0.005532005	0.831760347	0.002570848
0.007799562	0.496405751	0.003288278
0.006619723	0.158162773	0.493240476
0.973112285	0.479572564	0.504218996
0.168433383	0.992116511	0.252427965
0.498900533	0.990099669	0.250093192
0.842311382	0.993008554	0.759594560
0.518404186	0.030977042	0.750328124
0.391454339	0.650453210	0.566663682
0.338823825	0.593879759	0.690023005
0.338919789	0.781261742	0.061018020
0.335600644	0.527363479	0.057108108
0.716396987	0.788838983	0.062661067

0.716144562	0.540114522	0.060100492
0.959221601	0.792172670	0.056780219
0.958815634	0.536789000	0.056799501
0.533023417	0.852827430	0.130065814
0.663866162	0.204585060	0.559296846
0.664065361	0.455620706	0.556605041
0.273002177	0.181824297	0.556435466
0.265197009	0.435096025	0.559967756
0.035290871	0.187351644	0.551557243
0.023813454	0.441720009	0.557843089
0.467439324	0.153340012	0.624517381
0.201557592	0.325985432	0.307235211
0.451776773	0.328118414	0.311008066
0.209564611	0.702801108	0.309048921
0.462870806	0.704365075	0.310515732
0.207508266	0.944948018	0.306602418
0.463107556	0.945969284	0.305237621
0.143712655	0.515798509	0.378066063
0.800368428	0.667432070	0.809149027
0.544332385	0.657673180	0.805422187
0.798511505	0.286084086	0.810664713
0.548313737	0.281259030	0.814247251
0.800920963	0.038100190	0.813609242
0.550395370	0.040587038	0.809767842
0.865499675	0.473047495	0.877841353
0.672497272	0.794818342	0.946813405
0.669859946	0.544630826	0.943122149
0.295021921	0.789980114	0.946170688
0.292853713	0.537379503	0.942772329
0.052922875	0.791433752	0.948697865
0.051438384	0.535720110	0.948539436
0.480198324	0.852099776	0.876951754
0.328975052	0.192494586	0.445697457
0.338238418	0.447677940	0.449913710
0.710343838	0.199281901	0.443901420
0.713686049	0.450596243	0.441171885
0.957226455	0.198813438	0.440001160
0.955387235	0.450157076	0.444913149
0.523252904	0.130899757	0.376527220
0.792564034	0.330447644	0.695264280
0.541809261	0.331669599	0.698810160
0.809423089	0.722131550	0.697632790
0.555058360	0.733519793	0.695742130
0.808928311	0.961154997	0.702092230
0.554527581	0.976263881	0.697362959
0.844457030	0.527869582	0.630113780
0.215526819	0.658580065	0.194881156
0.469891012	0.661137342	0.196997508
0.207663640	0.282054573	0.191726789
0.456514120	0.281468958	0.193893149
0.204393059	0.038873181	0.197730646
0.459784627	0.038859945	0.196437016
0.144018397	0.465488911	0.124334902
0.144665435	0.852482975	0.128020570
0.530653954	0.465726584	0.126455784
0.853494763	0.141164437	0.626542211
0.136676669	0.133819312	0.371572673
0.523563981	0.520801663	0.379643291
0.864659607	0.859197319	0.882658601
0.477171928	0.470894665	0.874440968
0.455906153	0.538102567	0.628498197
0.288508385	0.536739826	0.695848286
0.379420280	0.606972098	0.721357703
0.443832874	0.706097603	0.561869919
0.383298546	0.609790981	0.534889638

THF@closed site

CONTCAR
1.0

12.6569995880	0.0000000000	0.0000000000
0.0000000000	12.6569995880	0.0000000000
0.0000000000	0.0000000000	26.3959999084

O	Si	Sn	C	H
130	63	1	8	16

Direct

0.325289428	0.653429747	0.067730740
0.345915049	0.812013268	0.001554826

0.238037780	0.839499176	0.085855506
0.448255569	0.818364978	0.086912178
0.346135944	0.504053175	0.996879637
0.228536263	0.470272779	0.077986084
0.438412756	0.477846473	0.084167011
0.713017464	0.660582304	0.074301556
0.838061154	0.828856051	0.062500246
0.651666820	0.847669899	0.105821393
0.838061392	0.494554371	0.062170055
0.645727456	0.470268667	0.099154145
0.965634048	0.662822127	0.060187303
0.029465824	0.841626346	0.101824030
0.025992984	0.484866679	0.103332676
0.682278335	0.333430052	0.566289783
0.674421251	0.173582658	0.500054777
0.742916167	0.139959306	0.593500972
0.541452289	0.174643919	0.575028539
0.680608630	0.484882802	0.496055573
0.758767545	0.523060799	0.585980177
0.548848867	0.501268148	0.571772277
0.256437004	0.298958600	0.546607554
0.152333573	0.128493458	0.562946260
0.345419526	0.138260469	0.601463854
0.151664063	0.476887703	0.563015044
0.337277651	0.437400550	0.606863678
0.029225737	0.307109416	0.562977433
0.952006161	0.124346077	0.587096989
0.963044405	0.488075972	0.605709016
0.326062948	0.326888561	0.322181225
0.188095793	0.335955709	0.245361090
0.152010262	0.211289540	0.322907388
0.138583958	0.421492338	0.332496792
0.472606242	0.327607185	0.250093132
0.506166637	0.221961170	0.335073858
0.504654944	0.432510793	0.334314257
0.337813199	0.700003266	0.317755342
0.170287162	0.821170628	0.313149154
0.157678217	0.627599716	0.353008568
0.507578611	0.822062969	0.311309725
0.515123785	0.636652529	0.357192248
0.339952946	0.949509561	0.311223030
0.160046920	0.011149241	0.352643192
0.527328849	0.013995010	0.345714837
0.676233590	0.653978586	0.813593984
0.841170609	0.674612463	0.750373304
0.840730727	0.771222472	0.838656366
0.860338032	0.561748385	0.832129955
0.517727017	0.673108280	0.747994542
0.499509454	0.755543172	0.839337707
0.495725602	0.549510121	0.826724172
0.674761593	0.312625855	0.816643238
0.821521044	0.160864532	0.818834603
0.864529014	0.354904503	0.849976897
0.529512167	0.160757452	0.826017439
0.486002594	0.350140989	0.854305506
0.673474610	0.008425384	0.815998793
0.859732509	0.975918353	0.858162284
0.480570525	0.963750362	0.844889045
0.672250926	0.670051634	0.931644797
0.661433995	0.809231758	0.007539644
0.783846855	0.846088529	0.928897560
0.574098706	0.855193019	0.919780314
0.670256197	0.519672275	0.002479064
0.772791088	0.487773508	0.916504920
0.562728405	0.494175732	0.918936670
0.298172474	0.662372172	0.934875548
0.174882799	0.829713285	0.942788482
0.366020381	0.847752392	0.903615236
0.171893835	0.495099068	0.940444291
0.358668178	0.481657475	0.897000670
0.045031320	0.662533581	0.944219470
0.986164212	0.842896342	0.902643859
0.981112003	0.480983704	0.903890610
0.359854460	0.310185373	0.443703055
0.323899746	0.132468417	0.500632703
0.221680865	0.165718853	0.415145248
0.429336369	0.122725606	0.416104764
0.322836190	0.473364800	0.504647434
0.249493226	0.480229408	0.411502421

0.453008682	0.495641053	0.427503020
0.703851759	0.325336993	0.431824982
0.834028840	0.161473334	0.435302705
0.638487637	0.137781262	0.402867228
0.841662884	0.485625803	0.431076318
0.651183248	0.511537790	0.397093087
0.973114729	0.322133809	0.434694082
0.019527698	0.138086796	0.392911434
0.042931095	0.512508154	0.412134677
0.668474555	0.298082978	0.690487146
0.835336566	0.315946221	0.751746655
0.861550748	0.249158606	0.657468319
0.816379547	0.449255764	0.676296234
0.508283556	0.311889023	0.755649388
0.472903967	0.244466409	0.664028764
0.521013081	0.449980050	0.680743992
0.680678189	0.720431268	0.689704537
0.848067462	0.841025949	0.688663840
0.861866772	0.645398498	0.651526153
0.513068736	0.851357996	0.696476698
0.497703195	0.677598476	0.646494627
0.679943919	0.973576128	0.693840802
0.862949550	0.039181426	0.662046254
0.499603420	0.037266854	0.649831235
0.342765689	0.667732298	0.187789589
0.184100315	0.648851156	0.254235744
0.157893509	0.757887840	0.170340016
0.175386831	0.547627628	0.167977586
0.495462656	0.645856917	0.256791741
0.523836851	0.769904196	0.177437648
0.519443095	0.560352564	0.167429075
0.332880795	0.283071011	0.177403241
0.166916952	0.157213464	0.192153409
0.143722966	0.344244897	0.148045033
0.499796361	0.159079686	0.191035599
0.523240805	0.351618946	0.154007941
0.333847761	0.029472662	0.190730155
0.150263578	0.965905011	0.154141411
0.513898611	0.971616507	0.148479089
0.006545307	0.829266012	0.002436894
0.007785534	0.496429384	0.003128192
0.008003791	0.156059489	0.492371738
0.978320599	0.471953094	0.506094754
0.173798382	0.989391804	0.252890408
0.499805868	0.985483110	0.247996047
0.842096746	0.991779685	0.758373797
0.514698148	0.028231123	0.749725640
0.365891010	0.645941377	0.568878651
0.315657973	0.568698883	0.688288093
0.339284062	0.780559659	0.060974941
0.335153669	0.526916087	0.057113171
0.717000067	0.786215425	0.062074136
0.716931581	0.536256790	0.059278101
0.959838569	0.790517628	0.056632381
0.959193408	0.535300314	0.057027116
0.533768117	0.851354003	0.129843816
0.660664201	0.207605496	0.558924854
0.663957000	0.460852712	0.556206286
0.271733612	0.173372507	0.553841472
0.269532979	0.424603432	0.555983186
0.037098970	0.181223482	0.550976038
0.030157324	0.435214758	0.559693277
0.465812176	0.148923963	0.623329401
0.201679736	0.324370831	0.306058019
0.452637851	0.326583833	0.310670972
0.211483806	0.699568808	0.309312701
0.464421898	0.701395273	0.310438752
0.211825386	0.942327797	0.307173520
0.467475146	0.942749083	0.303822458
0.147758871	0.509788156	0.377207726
0.803652287	0.665634692	0.808918476
0.548915863	0.658704817	0.806996465
0.799042106	0.285308629	0.808930218
0.550039709	0.283584714	0.811904728
0.798931420	0.035665907	0.812373757
0.549837768	0.040200546	0.808705389
0.867984712	0.471366197	0.875684440
0.672132909	0.794970810	0.946729422
0.670276105	0.543367267	0.942186058

0.296021760	0.788010001	0.946091831
0.293325275	0.535676301	0.942799807
0.053368632	0.790318072	0.948271453
0.051004607	0.534663677	0.948156178
0.481253296	0.854853272	0.876977265
0.333392709	0.184018657	0.444096744
0.345403910	0.437985152	0.446722835
0.712563515	0.199961290	0.442869782
0.719547927	0.451379806	0.440114468
0.957268775	0.195108816	0.439180851
0.959340274	0.447334886	0.446393430
0.525203705	0.126057819	0.375140488
0.793630183	0.328347325	0.693644047
0.542619824	0.329510212	0.696437716
0.807747841	0.719419360	0.694816649
0.553527474	0.728822470	0.694832504
0.806475163	0.960024536	0.701083422
0.551907778	0.972987473	0.696886837
0.849501967	0.525480151	0.629064858
0.215516448	0.655301094	0.194696024
0.469879597	0.660631716	0.196913257
0.207653850	0.278881788	0.191034615
0.456634998	0.279896706	0.193496346
0.206847951	0.035667747	0.197756335
0.461434603	0.036965098	0.194815308
0.143962651	0.462771833	0.124340847
0.144919351	0.850266337	0.128029346
0.530839801	0.465646178	0.126337409
0.853401780	0.139886364	0.624993443
0.139053911	0.131713584	0.370971918
0.529405296	0.517638266	0.378922105
0.866582572	0.858187199	0.882045746
0.477415979	0.470180452	0.874498785
0.448175699	0.536530614	0.627691984
0.433519840	0.703577161	0.533077478
0.357944489	0.772332549	0.501535892
0.247206584	0.748668373	0.523270011
0.271677375	0.709846199	0.576390624
0.288767099	0.480285674	0.721717775
0.185748875	0.513959169	0.746141076
0.202221662	0.633020401	0.754103065
0.272196680	0.666602969	0.709560573
0.362748563	0.752913475	0.461065531
0.208075911	0.685424626	0.501779556
0.290407717	0.775423169	0.602491081
0.476085722	0.643268585	0.511464834
0.489713490	0.750805914	0.555363715
0.211270377	0.658040941	0.592832148
0.195457771	0.818295896	0.523419678
0.377674550	0.856395304	0.505874395
0.171238199	0.471828580	0.781858563
0.242806420	0.647865653	0.790251911
0.227440715	0.705269039	0.679154932
0.352892786	0.470419228	0.749483705
0.283245087	0.409992546	0.697582424
0.337516695	0.717903674	0.721329510
0.127300039	0.676823735	0.754503489
0.118920140	0.498847753	0.720490217

o-Glu@closed site

CONTCAR
1.0

12.6569995880	0.0000000000	0.0000000000
0.0000000000	12.6569995880	0.0000000000
0.0000000000	0.0000000000	26.3959999084

O	Si	Sn	C	H
134	63	1	6	12

Direct

0.331647962	0.649960101	0.073460191
0.344357699	0.801210046	0.003548194
0.239411697	0.834804356	0.087815523
0.449593306	0.820321262	0.087750986
0.345880270	0.512605250	0.996933877
0.231158748	0.468460768	0.078579605
0.441351324	0.471419215	0.082893312
0.705534339	0.660894334	0.067556344
0.840483904	0.823750615	0.066508897

0.653306305	0.845857084	0.106403485
0.838909507	0.499315679	0.063598961
0.648418128	0.473923892	0.102095954
0.973928154	0.661829770	0.062485628
0.031924736	0.843999445	0.102275006
0.027941320	0.480444580	0.103406191
0.702116847	0.316056699	0.560349464
0.686134636	0.149399996	0.499907285
0.751014173	0.125128061	0.594693601
0.550162733	0.168519944	0.574505627
0.700357556	0.481792092	0.497216344
0.748124003	0.508362651	0.592181146
0.547401190	0.460310519	0.564617455
0.269975811	0.303165525	0.560115993
0.157251164	0.131375164	0.561597943
0.352018714	0.128103793	0.600971878
0.147818938	0.473487467	0.561989963
0.329264998	0.470449626	0.612755656
0.027082780	0.302617371	0.560580254
0.962014735	0.117341690	0.592567682
0.957305193	0.482198566	0.603393614
0.331045181	0.312850922	0.323190898
0.190461978	0.334750891	0.248177454
0.149226680	0.210923210	0.325767726
0.152499706	0.421225488	0.336403430
0.479617685	0.330499589	0.251727939
0.517549932	0.220592707	0.334515154
0.501068294	0.430802435	0.337969273
0.339323878	0.696532428	0.321446210
0.171403721	0.819988310	0.312295705
0.153939962	0.630135179	0.353789479
0.509211302	0.819529533	0.312714934
0.518953681	0.633629024	0.358248562
0.341368109	0.943809152	0.306573451
0.168052778	0.008451529	0.352578253
0.518204749	0.011618177	0.349120885
0.676431775	0.659689665	0.831102729
0.802315593	0.655126393	0.749836683
0.856231868	0.767094970	0.831054628
0.859722316	0.556621075	0.831238747
0.539689124	0.694881856	0.757843792
0.487535298	0.745093942	0.850303471
0.500018716	0.545131147	0.826834202
0.668992639	0.298291922	0.819146931
0.824069083	0.155890241	0.823182404
0.854277730	0.352757573	0.854708910
0.514007986	0.155642793	0.821210444
0.478558987	0.345378190	0.852096617
0.668470144	0.013151080	0.813640893
0.848543346	0.967404664	0.859702468
0.479580253	0.956393540	0.843622029
0.647085309	0.664759576	0.942325175
0.671102703	0.824268043	0.006821086
0.785711110	0.815570474	0.923382163
0.579122066	0.860533178	0.920166969
0.668684304	0.501363754	0.003790839
0.774230480	0.500363052	0.918449223
0.564738035	0.480181336	0.917987645
0.297211260	0.664708912	0.930998862
0.173404381	0.829757512	0.945431411
0.366451859	0.858727455	0.909676433
0.172089517	0.496347427	0.940965950
0.359485716	0.480824381	0.897754312
0.043555912	0.661945641	0.944624424
0.986950934	0.842052817	0.902192116
0.980310082	0.479244024	0.904372871
0.347925723	0.319306195	0.444349587
0.328333557	0.144532025	0.502103567
0.227199838	0.160681561	0.415935606
0.437198430	0.137528226	0.418545991
0.324042439	0.475659877	0.510160327
0.250520766	0.498227984	0.417386293
0.456031054	0.496508002	0.431318790
0.686801672	0.315630764	0.437217057
0.839102566	0.171718448	0.431706190
0.644830227	0.126886263	0.402145565
0.844768345	0.455421954	0.425641388
0.653375030	0.504682958	0.398922354
0.997752309	0.313387781	0.437402934

0.025482833	0.125319555	0.396313131
0.041819617	0.510540485	0.411416709
0.672109783	0.285012513	0.690642834
0.832211435	0.309416503	0.756024122
0.864443958	0.228504747	0.664251983
0.825131416	0.432435185	0.677771926
0.510808170	0.313848883	0.753580570
0.476357520	0.239622489	0.662607491
0.536265552	0.443148464	0.674631715
0.665020347	0.704239249	0.677516699
0.834509075	0.825348735	0.694486141
0.855832398	0.633208156	0.653641284
0.509984076	0.844434202	0.688877344
0.459771663	0.651235044	0.667764902
0.678536117	0.967582285	0.696421862
0.865710378	0.016866196	0.661434770
0.506158769	0.031634733	0.647812486
0.342190981	0.672966063	0.188730940
0.187468261	0.646067441	0.255476594
0.156084090	0.761430204	0.173434004
0.175545543	0.551251173	0.167494074
0.493578196	0.644347191	0.257515609
0.526499212	0.767614305	0.177889720
0.513161480	0.557852745	0.167874306
0.338017166	0.284892678	0.180691570
0.172010437	0.158677623	0.192931741
0.148798302	0.346342385	0.150640666
0.503774524	0.158979192	0.193492904
0.527718008	0.350331783	0.154742241
0.338134021	0.028871849	0.194465980
0.156139717	0.968394637	0.153523162
0.516953647	0.970504344	0.150481105
0.002260268	0.828091145	0.002689428
0.007312418	0.495826751	0.003430386
0.999776185	0.143353805	0.495629609
0.975833297	0.469613284	0.504483342
0.171290800	0.989962995	0.252502799
0.508302093	0.985372007	0.249912113
0.840938866	0.989282072	0.759622633
0.507020295	0.014900614	0.748182476
0.017694389	0.828626812	0.419423163
0.231707245	0.862391174	0.433510154
0.195351869	0.700829744	0.546527684
0.391820371	0.718336940	0.470580131
0.381239086	0.654243708	0.573774874
0.583806634	0.680695474	0.571901083
0.341087639	0.776428163	0.063394226
0.337756038	0.525642335	0.057769392
0.718061984	0.787909746	0.061324757
0.715905666	0.534362257	0.058754634
0.962176740	0.789054275	0.058034435
0.961428642	0.534806132	0.057788707
0.536003768	0.850531459	0.130694300
0.671697736	0.191002682	0.557983041
0.671831787	0.441872239	0.554363549
0.277398974	0.176628202	0.556703091
0.270470828	0.430313498	0.562488616
0.037625305	0.175686881	0.552516878
0.026743514	0.430783808	0.558298767
0.472235084	0.143436238	0.622420430
0.206219271	0.320319265	0.308550954
0.457244575	0.323946416	0.312105745
0.213788241	0.698716044	0.310197026
0.466134280	0.698618054	0.311869144
0.213897541	0.940555274	0.305272132
0.469263196	0.940249801	0.304265350
0.151260570	0.512806892	0.379413784
0.798281550	0.660468161	0.810740530
0.551218450	0.660588503	0.816029131
0.794865310	0.279140413	0.813103318
0.543600559	0.278209031	0.810319245
0.795192182	0.032840483	0.813581288
0.543041527	0.035379019	0.806524575
0.865544558	0.471776247	0.877292991
0.670516968	0.790685475	0.947428346
0.664358318	0.537370443	0.944703162
0.294665605	0.788145900	0.947239041
0.293147027	0.538653553	0.941985011
0.051777445	0.789711595	0.948850334

0.050449617	0.534101725	0.948316991
0.479685485	0.855558634	0.880932808
0.335581988	0.191707000	0.444992006
0.345246166	0.447013289	0.451272130
0.714925945	0.191068143	0.443283021
0.721202970	0.439350009	0.440418601
0.964147270	0.188951418	0.440709770
0.964270890	0.436575532	0.445208251
0.528919637	0.125986189	0.375936717
0.796796620	0.314276755	0.696915269
0.548021793	0.324279904	0.694586217
0.789133012	0.705094159	0.693654358
0.544153690	0.721422911	0.697962701
0.804241240	0.948586702	0.703731120
0.550713658	0.964819491	0.695555091
0.846723199	0.513328373	0.631261289
0.215437979	0.657379150	0.195555076
0.468878299	0.660261452	0.197385684
0.212563723	0.280395240	0.193291426
0.462611109	0.280699492	0.195505008
0.210297778	0.036931545	0.198187456
0.466205001	0.036477175	0.197076872
0.146367803	0.462955445	0.124953523
0.146376744	0.851146281	0.129296482
0.531493723	0.464416772	0.126606256
0.858871162	0.124035150	0.628507972
0.143379271	0.127364457	0.372561544
0.530836523	0.515626729	0.381989807
0.868479431	0.847634673	0.878856540
0.476316154	0.464657664	0.873827517
0.471868992	0.533270001	0.620187700
0.060824472	0.814786375	0.469027847
0.178422004	0.788719237	0.465406775
0.234718785	0.788303196	0.516521335
0.356640667	0.788289428	0.509399951
0.413684011	0.756106496	0.558168232
0.532116354	0.752575338	0.553187013
0.018771267	0.753077805	0.490641356
0.049834102	0.891002119	0.488457412
0.186751053	0.707951128	0.449182391
0.213746145	0.859629035	0.538325548
0.379147112	0.870718837	0.500609100
0.396925986	0.815867960	0.588074028
0.178660572	0.640574992	0.524630666
0.369629592	0.749144137	0.438318342
0.571420133	0.819049120	0.533268034
0.018010292	0.760607183	0.401951134
0.179289088	0.888909519	0.409148216
0.301611155	0.655058205	0.573076069

Glucopyranose@closed site

```
CONTCAR
   1.00000000000000
    12.6569995879999997    0.0000000000000000    0.0000000000000000
     0.0000000000000000   12.6569995879999997    0.0000000000000000
     0.0000000000000000    0.0000000000000000   26.3959999084000003
   O    Si   Sn   C    H
 134  63   1   6  12
Direct
  0.334962905  0.650947249  0.069581103
  0.345057523  0.808945380  0.002519249
  0.239830956  0.833638263  0.087484165
  0.450345949  0.822295633  0.086573614
  0.347170644  0.504750917  0.996861717
  0.233188041  0.470229407  0.079123911
  0.443849994  0.472082659  0.083091452
  0.708076897  0.662797659  0.067447865
  0.841217557  0.826788110  0.065012884
  0.654296933  0.848101240  0.105519360
  0.840699539  0.499922156  0.064827733
  0.650684216  0.475243177  0.102905398
  0.974023936  0.663639180  0.062713923
  0.032126479  0.846329464  0.102028858
  0.029924567  0.482025525  0.103785476
  0.687801880  0.326215549  0.563286528
  0.686192642  0.159926536  0.500891310
  0.753807357  0.136654130  0.594524510
```

0.550323576	0.167032957	0.575780740
0.703497395	0.483190204	0.495348158
0.755886470	0.517169445	0.590011617
0.554782407	0.494975202	0.562939117
0.273014596	0.306289932	0.560911051
0.158262098	0.134692340	0.561124642
0.351924112	0.129323502	0.601601461
0.147448547	0.475477761	0.558327956
0.323779676	0.476364978	0.611603798
0.027872850	0.304325237	0.560300810
0.964368152	0.119943151	0.593092813
0.962142016	0.484036125	0.603427440
0.330902820	0.311565013	0.321573085
0.187367023	0.333065246	0.247843505
0.147295352	0.212982592	0.326899512
0.154056150	0.423565221	0.335249681
0.483712607	0.328927011	0.252151466
0.517778102	0.222625549	0.336593971
0.499135231	0.432051493	0.338033338
0.341378033	0.700219745	0.319195793
0.171814743	0.821544623	0.312470455
0.158657019	0.630788755	0.354106416
0.512977666	0.821232675	0.311559758
0.521047945	0.635724693	0.356992858
0.342938144	0.944491065	0.307224952
0.171333392	0.010590542	0.353211359
0.521699278	0.012693831	0.348085421
0.677978369	0.660161592	0.829532351
0.807337036	0.657008352	0.749593483
0.857296343	0.767714130	0.831410665
0.861210466	0.556823272	0.831458900
0.544741830	0.672625758	0.752934267
0.490199126	0.751990455	0.841687460
0.500825261	0.546356063	0.828907934
0.670209125	0.304253235	0.819056306
0.823492874	0.158834157	0.822714484
0.857193459	0.353038958	0.855045904
0.518993603	0.157859699	0.823633692
0.479267337	0.346229430	0.853355966
0.671277871	0.012108780	0.815537494
0.854235202	0.969786745	0.858907893
0.479306635	0.961112819	0.844924844
0.650884441	0.664494566	0.941860187
0.670327058	0.825197005	0.006041217
0.784350211	0.821082765	0.922571512
0.575737387	0.855172473	0.918913203
0.671391660	0.502159039	0.004543443
0.773543420	0.498489094	0.918045777
0.563483153	0.480385179	0.920105547
0.297395929	0.662619514	0.934425177
0.174254294	0.830315774	0.944125386
0.364996089	0.851928543	0.906109772
0.171902892	0.494339773	0.941248005
0.358336180	0.480884056	0.897500991
0.045170044	0.662188885	0.944435435
0.987582100	0.842641578	0.902231593
0.980738678	0.479703987	0.904927020
0.357889632	0.318994898	0.443170793
0.330134797	0.147824537	0.502560304
0.225968592	0.165426384	0.417015630
0.434160703	0.131089215	0.418145370
0.330052316	0.475297430	0.509173219
0.252410823	0.494234600	0.416783372
0.457537147	0.502560663	0.431038330
0.688722365	0.317730144	0.434634322
0.838091529	0.169125072	0.431627258
0.642865607	0.126302800	0.404050003
0.846195665	0.457296216	0.422954835
0.654127905	0.507115661	0.397515866
0.995611392	0.312103703	0.435882775
0.025355244	0.122155592	0.396762055
0.044617684	0.509007109	0.411108759
0.672568606	0.289195113	0.691789833
0.833579788	0.314974822	0.756226536
0.866684888	0.232046720	0.665358865
0.824367466	0.435916971	0.676410075
0.509511933	0.313943831	0.754781527
0.477814460	0.235686251	0.664176254
0.529764376	0.440132614	0.674800771

0.675113164	0.714275703	0.675984984
0.844616057	0.829658423	0.695537128
0.863769601	0.636586126	0.653812062
0.512689039	0.843787198	0.696381176
0.476550416	0.660978557	0.658340889
0.680807326	0.965450182	0.696164652
0.865062699	0.020484272	0.660443902
0.505297292	0.028309505	0.649035521
0.342826629	0.672910197	0.188796826
0.186778955	0.647742145	0.255615685
0.155822504	0.760210155	0.172750836
0.176896906	0.550214974	0.168413355
0.497066841	0.646324458	0.256814571
0.527123558	0.767705054	0.176352382
0.514359114	0.557744924	0.168212200
0.338933105	0.286235895	0.181974205
0.172939571	0.158170580	0.191700225
0.150289747	0.345728933	0.149878209
0.504761509	0.159260872	0.192553510
0.529047505	0.350319810	0.154892108
0.339188946	0.028806231	0.193958953
0.156817060	0.967513219	0.153769799
0.518016344	0.971273712	0.149750096
0.004875144	0.829120847	0.002454157
0.007847310	0.497152333	0.003935588
-0.001094032	0.144230450	0.495722447
0.971224096	0.467306887	0.503463557
0.172440609	0.991684408	0.252913457
0.509044202	0.986517830	0.249396206
0.842756733	0.994565781	0.759140070
0.512217470	0.021310142	0.749292025
0.189736090	0.788055494	0.651973249
0.391842836	0.672963375	0.565689216
0.157225819	0.839409909	0.551379328
0.250536188	0.856870255	0.455494885
0.480263102	0.818409922	0.451167314
0.523070341	0.805372721	0.552785181
0.342227481	0.778343723	0.061917141
0.340193418	0.524725797	0.057377259
0.718899672	0.790076123	0.060501700
0.718082847	0.535809598	0.059629141
0.963040675	0.791046198	0.057666764
0.962798034	0.536159748	0.058461846
0.536740148	0.851613727	0.129630879
0.669559986	0.198643556	0.558992587
0.674615771	0.453300732	0.553568827
0.278409053	0.178979513	0.557143408
0.269112937	0.433883054	0.560013516
0.038540474	0.177505711	0.552116950
0.026670664	0.432251717	0.556645424
0.472135013	0.140166810	0.623320842
0.205316001	0.320872914	0.308249240
0.457521886	0.324016297	0.312201905
0.214854441	0.700453248	0.310233174
0.468397074	0.701141614	0.310929744
0.215000500	0.941579490	0.305835235
0.470994996	0.940982958	0.303905698
0.153422883	0.513862200	0.378992801
0.800759800	0.660772519	0.810642224
0.553527201	0.658255747	0.813297554
0.795870973	0.282579459	0.813068345
0.544914923	0.280336656	0.811572424
0.797792243	0.034934891	0.813649115
0.546241511	0.037729741	0.808220771
0.866676026	0.471980822	0.877619987
0.670449475	0.791158676	0.946701115
0.665284915	0.536906569	0.945369519
0.294918590	0.787914181	0.946851942
0.293030144	0.535803575	0.942877943
0.053008800	0.790124352	0.948433658
0.051134473	0.534226403	0.948738793
0.478766579	0.854214107	0.878304621
0.336948606	0.192264662	0.444984721
0.348705398	0.446438265	0.449969176
0.714691575	0.193426735	0.443113492
0.723128814	0.441190898	0.438082581
0.963210271	0.187531654	0.440352160
0.964854672	0.436234142	0.443583679
0.528463194	0.125197673	0.376419638

0.797804060	0.318203705	0.697159043
0.546883772	0.322114837	0.695952632
0.798871653	0.709854262	0.693876244
0.553527037	0.722075859	0.696740862
0.807331911	0.951665906	0.703592479
0.552555959	0.965235634	0.697467354
0.852120679	0.517366092	0.630866794
0.215829890	0.657385928	0.195929048
0.469959333	0.660952880	0.196992993
0.212814000	0.280029186	0.193044936
0.463948711	0.280842684	0.195748856
0.211472614	0.036766152	0.198117094
0.467392171	0.036844838	0.196423907
0.148192823	0.463284484	0.125263564
0.146825358	0.850642849	0.129017794
0.533554470	0.464544570	0.127075588
0.860958039	0.128936017	0.628537923
0.143699447	0.128329028	0.373290947
0.531568168	0.518558714	0.380770883
0.869754074	0.849683017	0.878651598
0.476470711	0.465188572	0.875278139
0.466129924	0.529645916	0.621621483
0.466797612	0.720833949	0.530790689
0.310593208	0.748054954	0.583561023
0.245454748	0.775928570	0.536543574
0.314992934	0.833366583	0.498576675
0.409284471	0.764122832	0.484035349
0.241431127	0.703657326	0.626428802
0.519387935	0.655307839	0.520281303
0.216929604	0.701171822	0.519423030
0.343544074	0.907465816	0.516190802
0.379488377	0.696252607	0.462249539
0.351445335	0.818098415	0.598711249
0.290676763	0.664812785	0.655053388
0.147088551	0.825041444	0.626619916
0.126507746	0.867937065	0.520321712
0.293769803	0.896198623	0.431436329
0.523316850	0.864692078	0.472510743
0.579399116	0.777980770	0.574284078
0.184426148	0.645441216	0.611580510
